# What is the optimal biological therapy for moderate to severe ulcerative colitis: a systematic review and network meta-analysis

**DOI:** 10.3389/fphar.2025.1602024

**Published:** 2025-07-11

**Authors:** Longbin Huang, Chen Kong, Ningning Yue, Hailan Zhao, Yuan Zhang, Chengmei Tian, Zhiliang Mai, Daoru Wei, Ruiyue Shi, Jun Yao, Lisheng Wang, Defeng Li

**Affiliations:** ^1^ Department of Gastroenterology, Shenzhen People’s Hospital, The Second Clinical Medical College, Jinan University, Shenzhen, Guangdong, China; ^2^ Department of Medical Administration, Huizhou Institute of Occupational Diseases Control and Prevention, Huizhou, Guangdong, China; ^3^ Department of Emergency, Shenzhen People’s Hospital (The Second Clinical Medical College, Jinan University, The First Affiliated Hospital, Southern University of Science and Technology), Shenzhen, Guangdong, China; ^4^ Department of Rehabilitation, Shenzhen People’s Hospital (The Second Clinical Medical College, Jinan University, The First Affiliated Hospital, Southern University of Science and Technology), Shenzhen, Guangdong, China; ^5^ Department of Gastroenterology, Shenzhen People’s Hospital (The Second Clinical Medical College, Jinan University, The First Affiliated Hospital, Southern University of Science and Technology), Shenzhen, Guangdong, China

**Keywords:** ulcerative colitis, biological therapy, clinical trials, bayesian network meta-analysis, systematic review

## Abstract

**Objective:**

The biologics for moderate to severe ulcerative colitis (UC) have expanded with an increasing array. We performed an updated network meta-analysis to evaluate and compare the relative efficacy and safety profiles of biologics in moderate to severe UC.

**Design:**

We searched literature to 18 May 2024, to identify eligible studies. The clinical remission, clinical response, or endoscopic improvement, stratified by previous exposure or naive to biologics, and safety were assessed. A network meta-analysis was performed through the bayesian model, obtaining pairwise relative ratios (RR) and 95% confidence intervals (CI). The surface under the cumulative ranking probabilities (SUCRA) was used to rank the included agents for each outcome.

**Results:**

A total of 23 trials (10,839 patients) were included. In induction therapy, based on achieving clinical remission and endoscopic improvement, infliximab 5 mg/kg ranked first. For clinical response, ustekinumab 6 mg/kg superior to other drugs. Infliximab 5 mg/kg demonstrated superior efficacy in biologic-naive patients, whereas ustekinumab 6 mg/kg was the most effective in biologic-exposed patients. No significant differences between active interventions were observed when assessing safety outcomes, except for visilizumab. In maintenance therapy, for clinical remission and endoscopic improvement, vedolizumab 108 mg every other week and vedolizumab 300 mg every 4 weeks ranked first respectively, with infliximab 5 mg/kg performed best in achieving clinical response. Regarding safety ranking, golimumab 100 mg was the lowest.

**Conclusion:**

In this network meta-analysis, infliximab and vedolizumab emerged as the most effective biologics for inducing and maintaining efficacy outcomes for patients with UC. Most drugs were found to be safe and well-tolerated, with ustekinumab and mirikizumab exhibiting particularly favorable safety profiles.

## Introduction

Ulcerative colitis (UC) is a chronic, idiopathic, and potentially disabling inflammatory bowel disease characterized by relapsing and remitting episodes of bloody stool, abdominal pain, and tenesmus. In 2023, the prevalence of UC was estimated to be 5 million cases around the world, and the incidence is increasing worldwide (Le Berre et al., 2023). The pathogenesis of UC remains complex and not fully elucidated. It is generally believed that the initial stages of pathogenesis underpinned by a disruption of the gut barrier and a loss of mucosal homeostasis (Neurath, 2014). Epithelial barrier defects, dysregulated immune responses, and dysbiosis are integral in this interplay of initiating and perpetuating inflammation.

Conventional therapies primarily include pharmacological treatments, surgical interventions, and lifestyle modifications. Pharmacological treatment remains the cornerstone of therapy, 5-aminosalicylates demonstrated efficacy in managing mild to moderate disease activity, and corticosteroids will be used in the more severe flares, and immunosuppressants (Barberio et al., 2021; Ford et al., 2011; Ford et al., 2012a; Ford et al., 2012b). However, traditional treatments have limitations, especially corticosteroids, which may lead to serious adverse effects with long-term use and dependent on them or develop resistance to these drugs, while the variable efficacy and potential infectious risks associated with immunosuppressive agents (Ford et al., 2012b; Faubion et al., 2001; Ho et al., 2006).

Given the limitations of conventional therapies, the advent of biologics has revolutionized the management of UC by targeting specific immune pathways. Over the past 2 decades, the emergence of anti-tumor necrosis factor biologics, such as infliximab, adalimumab, and golimumab, has significantly transformed the treatment landscape of UC. These therapies target specific immune pathways to mitigate inflammation, thereby ameliorate symptoms and inducing clinical remission (Liu et al., 2015). Biologics with other targets were later approved for the treatment of moderate to severe UC, such as anti-integrin antibodies (e.g., vedolizumab or etrolizumab), and IL-12/23 inhibitors (e.g., ustekinumab) have greatly enhanced treatment effects for moderate to severe UC (Rutgeerts et al., 2013; Feagan et al., 2005; Sands et al., 2019). However, this field is rapidly evolving, and several novel agents have already demonstrated efficacy in recent phase Ⅲ clinical trials. These drugs were not included in the most recent network meta-analyses.

Currently, the variety of medications available for ulcerative colitis is steadily growing. The effectiveness and safety of these drugs have become significant factors that clinicians and patients consider when choosing biologics. However, directly comparing these treatments are not very feasible, network meta-analysis offers an indirect comparison that can serve as a useful reference. Therefore, to provided clinicians with more authoritative and efficient guidelines, an updated and comprehensive network meta-analysis was conducted to evaluate the effectiveness and safety of biological therapy that have progressed to phase Ⅲ trials. In particular, we compared these therapies with each other and with placebo regarding their ability to induce and maintain remission, elicit clinical response, and achieve endoscopic improvement in patients with moderate to severe UC.

## Methods

### Search strategy and selection criteria

A prior established protocol was used to conduct this study, and report according to the Preferred Reporting Items for Systematic Reviews and Meta-Analyses extension statement for systematic reviews incorporating network meta-analyses for healthcare interventions (Moher et al., 2009).

Two independent investigators (Huang and Kong) conducted literature searches. We searched PubMed, Embase, Web of Science, the Cochrane central register of controlled trials (from the establishment of the database to 18 May 2024). In addition, we also searched ClinicalTrials.gov for recently completed clinical trials. There were no language restrictions. We included phase Ⅲ randomized controlled trials (RCTs) that met the following inclusion criteria: (1) Studies which included adult patients with moderate to severe UC (defined as a mayo score of 6–12, with an endoscopic subscore of 2–3) who were either biologic-naive or have been previously exposed to at least one biologic (Schroeder et al., 1987). (2) Studies assessing the following biological agents: infliximab, adalimumab, golimumab, etrolizumab, vedolizumab, ustekinumab, mirikizumab, visilizumab, guselkumab, ontamalimab, eldelumab, basiliximab, daclizumab, certolizumab pegol and natalizumab. (3) Biological agents compared with each other or with placebo.

In addition, studies included in the analysis were required to provide sufficient data on efficacy and safety. Consequently, phase Ⅱ clinical trials were excluded because their typically smaller sample sizes, which may lead to either overestimation or underestimation of treatment effects. Furthermore, as phase Ⅱ trials primarily focus on dose-ranging exploration, some doses examined might not be further assessed for therapeutic efficacy.

Two investigators (Huang and Kong) independently appraised all the abstracts retrieved through the search. Subsequently, we conducted a more detailed evaluation employing pre-designed templates. We resolved disagreements between investigators by discussion. Our study protocol was registered with PROSPERO (No. CRD42025643026).

### Outcome assessment

Two main analyses were carried out: induction therapy and maintenance therapy for UC. Efficacy metrics were clinical remission (defined as mayo score ≤2, with no subscore >1), clinical response (defined as a mayo score ≥3 points lower and ≥30% lower than baseline, rectal bleeding subscore ≥1 point or ≤1), endoscopic improvement (defined as a mayo endoscopic subscore of 0 or 1) (Lewis et al., 2008). All efficacy outcomes were evaluated uniformly according to the standardized Mayo score definition, with assessments conducted between weeks 6 and 14 for induction therapy and at week 30–66 for maintenance therapy. The endpoint criteria utilized in each trial are summarized in Supplementary Table S1. For safety evaluation, we assessed the number of patients who received at least one dose of the study drug. The safety assessed included adverse events (total numbers of patients who face adverse events), as well as serious adverse events, infections and adverse events leading to study withdrawal, if reported. The safety analysis results were stratified by treatment stage: the induction period (<14 weeks) and the maintenance period (≥14 weeks). An additional exploratory analysis was conducted to assess the efficacy of induction therapy in both biologic-naive and biologic-experienced populations.

### Data extraction

After reaching consensus on eligibility, two investigators (Huang and Kong) independently extracted data from all eligible studies into a standardized Microsoft Excel spreadsheet, recording dichotomous outcomes (clinical remission or not, endoscopic improvement or not, clinical response or not). We extracted these data from each trial, where available: relevant publication information (i.e., author, title, year, journal), country of origin, the number of centers, patient characteristics (e.g., age, sex), disease extent and duration, proportion of biologic-naive patients), dose and treatment schedule of biologics agents and placebo, and duration of follow up. Patients who dropped out were considered treatment failures (no response to biological therapy or placebo) when assessing efficacy, as permitted by trial reporting. Data extracted by two investigators were compared, and any discrepancies were resolved through discussion.

### Quality assessment and risk of bias

In order to ensure that the inclusion of the trials under examination was of a uniformly high caliber, we used the Cochrane risk of bias tool to assess the quality of included trials (Higgins et al., 2024). Two authors (Huang and Kong) performed the evaluation independently, resolving disagreements by discussion. We evaluated the methods used to generate the randomization schedule and conceal treatment allocation. We also assessed whether blinding was implemented for patients, staff and outcome assessment, whether there was evidence of incomplete outcome data, and whether there was evidence of selective reporting of outcomes.

### Data synthesis and statistical analysis

All statistical analyses in this study were conducted by R software (version 4.4.2) and Stata software (version 14.2). We conducted this network meta-analysis utilizing the “GEMTC” and the “JAGS” package in R Studio to perform Bayesian network meta-analysis, which integrates both direct and indirect comparisons (Shim et al., 2019). A Markov Chain Monte Carlo simulation technique was used to perform the network meta-analysis (Neupane et al., 2014). Annealing times were set at 20,000 iterations, with 50,000 simulation iterations, and thinning interval of 1. Deviance information criteria (DIC) was used to compare and consider the fixed and random effect models (Dias et al., 2010). Furthermore, when a closed loop is formed among various intervention measures, the node splitting technique was applied for network consistency assessment and if the P value is greater than 0.05, it is considered that there is no significant difference (van Valkenhoef et al., 2016). We assessed the convergence adequacy (achieving a stable equilibrium state) through the visually inspecting the trace plots and estimating the values of the Brooks–Gelman–Rubin statistic (Brooks and Gelman, 1998). Once convergence was confirmed, the posterior distributions of the model parameters were derived. The probabilities of all treatment regimens occupying each ranking position were calculated. The rankings of the treatment regimens were then compared using the surface under the cumulative ranking curve (SUCRA) (Salanti et al., 2011). A higher SUCRA score indicates superior efficacy or safety. We performed the study according to the Preferred Reporting Items for Systematic Reviews and Meta-analyses extension statement for network meta-analyses, to explore direct and indirect treatment comparisons of the efficacy and safety of each intervention (Hutton et al., 2015).

The network plots for comparisons among distinct regimens were depicted by Stata that node size corresponding to number of study subjects, and connection size corresponding to number of studies. Heterogeneity among studies was evaluated by means of I^2^ tests, with values greater than 50% suggesting substantial heterogeneity. We used the pooled relative ratios (RR), with their corresponding 95% confidence intervals (CI) to judge efficacy of each comparison tested, using a random effects model as a conservative estimate, allowing for any heterogeneity among studies. As there were direct comparisons among some active therapies, we were able to perform consistency modelling to check the correlation between direct and indirect evidence across the network (Higgins et al., 2012).

## Results

The search strategy yielded 5182 citations, and 143 of these were initially identified as potentially relevant and obtained for further evaluation. From this pool, we excluded 123 studies that did not meet the eligibility criteria, with detailed reasons outlined in Supplementary Figure S1. Consequently, the study included 20 eligible articles, which reported findings from 23 RCTs with 10,839 UC patients (Reinisch et al., 2011; Sandborn et al., 2012; Sandborn et al., 2010; Suzuki et al., 2014; Sandborn et al., 2020; Peyrin-Biroulet et al., 2022; Rubin et al., 2022; Danese et al., 2022; Panés et al., 2022; Rutgeerts et al., 2005; Jiang et al., 2015; D'Haens et al., 2023; Sandborn et al., 2014a; Sandborn et al., 2014b; Sands et al., 2019a; Feagan et al., 2013; Motoya et al., 2019; Sands et al., 2019b; Hibi et al., 2017; Vermeire et al., 2022). Detailed characteristics of the individual RCT was presented in Supplementary Table S2. 15 RCTs were assessed as having a low risk of bias across all domains, which was summarized in Supplementary Figure S2 (Sandborn et al., 2010; Suzuki et al., 2014; Peyrin-Biroulet et al., 2022; Rubin et al., 2022; Danese et al., 2022; Jiang et al., 2015; D'Haens et al., 2023; Sandborn et al., 2014a; Sandborn et al., 2014b; Sands et al., 2019b; Feagan et al., 2013; Motoya et al., 2019; Vermeire et al., 2022). Notably, after a comprehensive retrieval and screening process, we identified the following drugs-ontamalimab, eldelumab, basiliximab, guselkumab, daclizumab, certolizumab pegol, and natalizumab-lacked eligible trials.

In addition, to search for any recent updates of RCTs, we re-examined the 8 biological agents included in this article in the same databases from 19 May 2024 to 10 May 2025 using the original search strategy. This updated search yielded 699 new citations, from which we identified 2 additional relevant studies (Naganuma et al., 2025; Talar-Wojnarowska et al., 2022). After careful evaluation, we found that these new findings did not substantially differ from our original conclusions and therefore do not significantly impact the existing evidence synthesis. Therefore, we have decided not to re-analyze the dataset at this stage, we propose addressing these updates in future work.

The consistency test and convergence assessment demonstrated that the models for each outcome indicator achieved satisfactory convergence, as evidenced by potential scale reduction factor (PSRF) values approaching 1 (Supplementary Figure S3). In terms of inconsistency, after conducting the test, all outcomes showed no statistically significant difference between direct and indirect comparisons (P > 0.05), except for the clinical response to maintenance therapy and the subset of biologic-exposed patients which did not form a closed loop.

### Clinical remission

**FIGURE 1:**
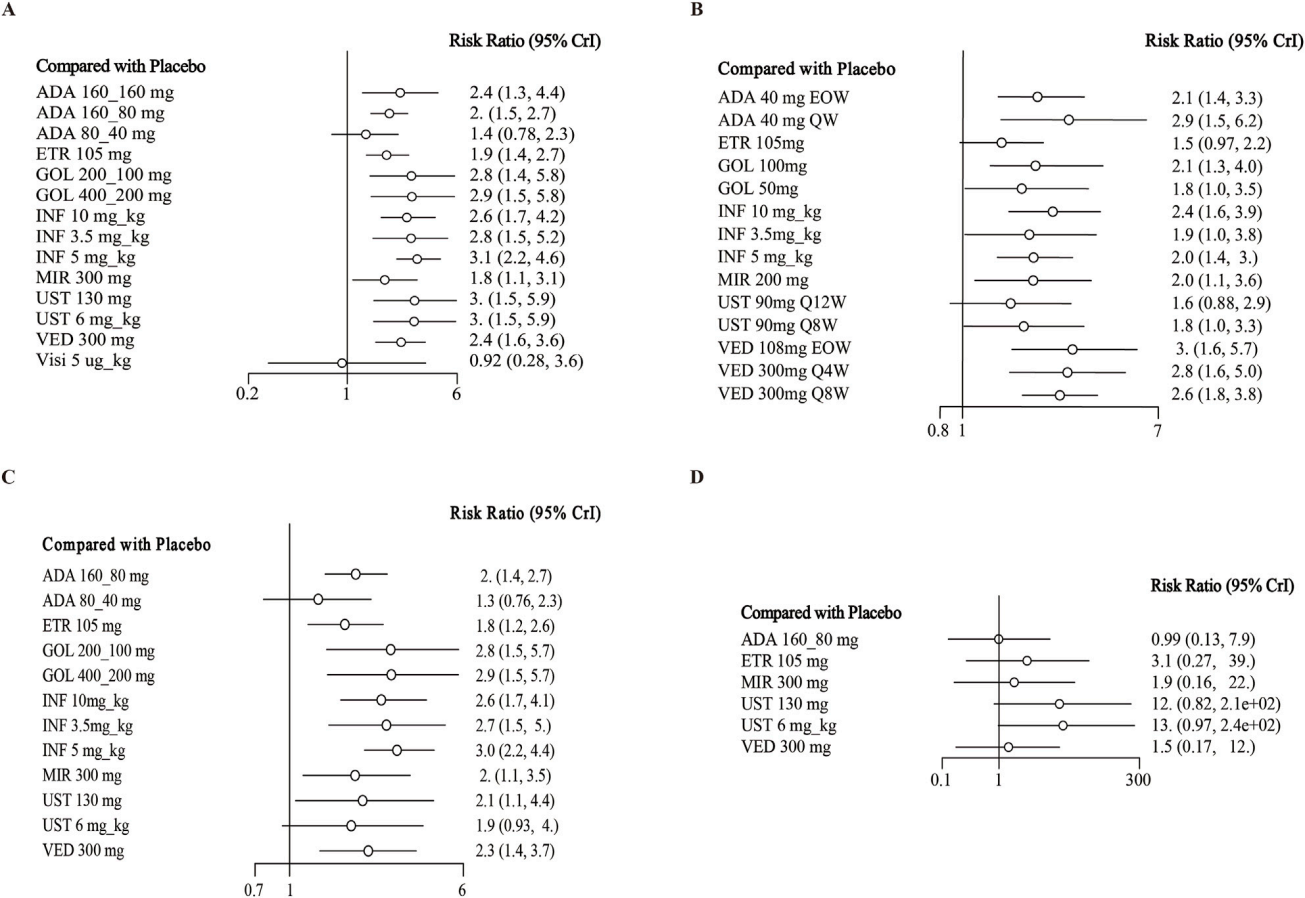
Forest plot for achieving clinical remission in **(A)** induction therapy: all patients, **(B)** maintenance therapy: all patients, **(C)** biologic-naive patients, **(D)** biologic-exposed patients. Note: ADA, adalimumab; ETR, etrolizumab; GOL, golimumab; IFX, infliximab; UST, ustekinumab; VED, vedolizumab; MIR, mirikizumab; Visi, visilizumab; EOW, every other week; QW, every week; Q12W, every 12 weeks; Q8W, every 8 weeks; Q4W, every 4 weeks

In the evaluation of induction therapy for clinical remission, data from 18 RCTs with low heterogeneity (I^2^ = 0%) were analyzed (Reinisch et al., 2011; Sandborn et al., 2012; Sandborn et al., 2010; Suzuki et al., 2014; Peyrin-Biroulet et al., 2022; Rubin et al., 2022; Danese et al., 2022; Panés et al., 2022; Rutgeerts et al., 2005; Jiang et al., 2015; D'Haens et al., 2023; Sandborn et al., 2014; Sands et al., 2019; Feagan et al., 2013; Motoya et al., 2019; Sands et al., 2019). The network plot is provided in Supplementary Figure S4. When data were pooled, except for adalimumab 80/40 mg and visilizumab 5 μg/kg, other drugs were superior to placebo (Figure 1A). In indirect comparisons, adalimumab 160/80 mg, adalimumab 80/40 mg and etrolizumab 105 mg showed inferior efficacy compared to infliximab 5 mg/kg (Table 1). When comparing active treatments, the ranking of probabilities by SUCRA indicated that infliximab 5 mg/kg was the most effective drug among all interventions (SUCRA 0.811). Ustekinumab 130 mg ranked second (SUCRA 0.731), followed by ustekinumab 6 mg/kg (SUCRA 0.726) and golimumab 400/200 mg (SUCRA 0.697) (Supplementary Figure S5).

**TABLE 1:** League table for achieving clinical remission and endoscopic improvement of the induction phase: all patients.

ADA 160/160 mg	0.88 (0.68, 1.13)	0.71 (0.5, 1.01)	0.94 (0.69, 1.29)	0.99 (0.66, 1.5)	1.06 (0.71, 1.6)	1.27 (0.89, 1.81)	1.3 (0.78, 2.15)	1.29 (0.92, 1.82)	1.04 (0.72, 1.5)	0.67 (0.5, 0.91)	1.28 (0.81, 2.05)	1.32 (0.83, 2.11)	0.98 (0.65, 1.47)	0.76 (0.39, 1.65)	Endoscopic Improvement
19 (0.7, 2.05)	ADA 160/80 mg	0.81 (0.64, 1.02)	1.07 (0.89, 1.3)	1.13 (0.82, 1.57)	1.21 (0.89, 1.67)	1.44 (1.13, 1.85)	1.48 (0.95, 2.29)	1.47 (1.18, 1.84)	1.18 (0.91, 1.55)	0.77 (0.66, 0.89)	1.45 (1, 2.17)	1.5 (1.03, 2.22)	1.12 (0.82, 1.53)	0.86 (0.47, 1.81)	
1.75 (0.84, 3.78)	1.47 (0.88, 2.51)	ADA 80/40 mg	1.32 (1, 1.76)	1.39 (0.96, 2.03)	1.49 (1.03, 2.16)	1.78 (1.3, 2.43)	1.82 (1.12, 2.96)	1.81 (1.35, 2.45)	1.46 (1.05, 2.02)	0.94 (0.74, 1.2)	1.8 (1.17, 2.8)	1.85 (1.2, 2.88)	1.38 (0.96, 1.98)	1.06 (0.56, 2.26)	
1.25 (0.64, 2.33)	1.05 (0.72, 1.48)	0.71 (0.38, 1.27)	ETR 105 mg	1.05 (0.75, 1.48)	1.13 (0.81, 1.58)	1.35 (1.06, 1.71)	1.38 (0.88, 2.1)	1.37 (1.12, 1.68)	1.1 (0.83, 1.47)	0.72 (0.6, 0.85)	1.36 (0.92, 2.04)	1.4 (0.95, 2.09)	1.04 (0.75, 1.44)	0.8 (0.43, 1.7)	
0.83 (0.33, 2.06)	0.7 (0.32, 1.45)	0.48 (0.2, 1.11)	0.67 (0.31, 1.44)	GOL 200/100 mg	1.07 (0.83, 1.37)	1.28 (0.9, 1.84)	1.31 (0.78, 2.13)	1.3 (0.93, 1.84)	1.05 (0.73, 1.5)	0.68 (0.51, 0.9)	1.29 (0.82, 2.04)	1.34 (0.84, 2.1)	0.99 (0.67, 1.47)	0.76 (0.4, 1.67)	
0.83 (0.33, 2.03)	0.69 (0.32, 1.43)	0.47 (0.2, 1.1)	0.66 (0.31, 1.42)	0.99 (0.57, 1.73)	GOL 400/200 mg	1.2 (0.84, 1.7)	1.22 (0.74, 2)	1.22 (0.87, 1.71)	0.98 (0.69, 1.4)	0.64 (0.48, 0.83)	1.2 (0.77, 1.91)	1.25 (0.79, 1.95)	0.92 (0.62, 1.37)	0.71 (0.37, 1.56)	
0.9 (0.41, 1.84)	0.76 (0.44, 1.24)	0.51 (0.25, 1)	0.72 (0.44, 1.17)	1.08 (0.47, 2.43)	1.09 (0.48, 2.45)	INF 10 mg/kg	1.02 (0.66, 1.56)	1.02 (0.86, 1.21)	0.82 (0.6, 1.11)	0.53 (0.43, 0.65)	1.01 (0.67, 1.54)	1.04 (0.69, 1.57)	0.77 (0.55, 1.09)	0.6 (0.32, 1.25)	
0.84 (0.36, 1.99)	0.7 (0.36, 1.39)	0.48 (0.21, 1.09)	0.67 (0.36, 1.32)	1.01 (0.41, 2.57)	1.02 (0.41, 2.56)	0.93 (0.5, 1.85)	INF 3.5 mg/kg	1 (0.67, 1.5)	0.8 (0.5, 1.29)	0.52 (0.34, 0.79)	0.99 (0.57, 1.72)	1.02 (0.59, 1.76)	0.75 (0.46, 1.26)	0.58 (0.28, 1.35)	
0.76 (0.37, 1.48)	0.64 (0.4, 0.96)	0.43 (0.22, 0.79)	0.61 (0.41, 0.9)	0.91 (0.42, 1.98)	0.91 (0.42, 1.98)	0.84 (0.58, 1.23)	0.9 (0.5, 1.53)	INF 5 mg/kg	0.81 (0.6, 1.07)	0.52 (0.43, 0.62)	0.99 (0.67, 1.49)	1.02 (0.68, 1.52)	0.76 (0.55, 1.05)	0.58 (0.31, 1.23)	
1.28 (0.57, 2.88)	1.08 (0.58, 1.97)	0.73 (0.34, 1.54)	1.03 (0.56, 1.96)	1.54 (0.66, 3.7)	1.56 (0.67, 3.71)	1.43 (0.74, 2.92)	1.53 (0.69, 3.41)	1.7 (0.92, 3.27)	MIR 300 mg	0.65 (0.52, 0.81)	1.23 (0.81, 1.88)	1.27 (0.84, 1.93)	0.94 (0.66, 1.34)	0.73 (0.38, 1.55)	
2.37 (1.28, 4.4)	1.99 (1.47, 2.69)	1.35 (0.78, 2.3)	1.9 (1.37, 2.73)	2.85 (1.45, 5.76)	2.87 (1.47, 5.75)	2.64 (1.74, 4.2)	2.83 (1.52, 5.15)	3.13 (2.24, 4.58)	1.84 (1.09, 3.13)	Placebo	1.9 (1.34, 2.74)	1.96 (1.38, 2.8)	1.46 (1.11, 1.92)	1.12 (0.62, 2.31)	
0.79 (0.32, 1.95)	0.66 (0.31, 1.38)	0.45 (0.19, 1.06)	0.63 (0.3, 1.38)	0.95 (0.37, 2.48)	0.96 (0.37, 2.48)	0.88 (0.4, 2.02)	0.94 (0.38, 2.35)	1.04 (0.49, 2.29)	0.62 (0.26, 1.44)	0.33 (0.17, 0.65)	UST 130 mg	1.03 (0.77, 1.38)	0.77 (0.49, 1.19)	0.59 (0.29, 1.36)	
0.8 (0.32, 1.98)	0.67 (0.31, 1.39)	0.46 (0.19, 1.06)	0.64 (0.3, 1.37)	0.95 (0.37, 2.49)	0.96 (0.37, 2.5)	0.88 (0.4, 2.01)	0.95 (0.38, 2.35)	1.05 (0.5, 2.28)	0.62 (0.26, 1.44)	0.34 (0.17, 0.65)	1.01 (0.58, 1.74)	UST 6 mg/kg	0.74 (0.48, 1.16)	0.57 (0.28, 1.32)	
98 (0.52, 1.95)	0.82 (0.57, 1.23)	0.56 (0.3, 1.05)	0.79 (0.5, 1.33)	1.18 (0.54, 2.71)	1.19 (0.55, 2.7)	1.09 (0.63, 2.06)	1.18 (0.57, 2.42)	1.3 (0.79, 2.28)	0.76 (0.4, 1.52)	0.42 (0.28, 0.63)	1.25 (0.57, 2.83)	1.24 (0.57, 2.77)	VED 300 mg	0.77 (0.4, 1.65)	
2.59 (0.59, 9.98)	2.16 (0.53, 7.4)	1.46 (0.35, 5.42)	2.07 (0.52, 7.23)	3.08 (0.67, 12.6)	3.1 (0.7, 12.64)	2.87 (0.69, 10.55)	3.08 (0.69, 11.61)	3.41 (0.85, 2.07)	2.01 (0.48, 7.49)	1.09 (0.28, 3.63)	3.26 (0.7, 13.41)	3.23 (0.72, 3.09)	2.61 (0.63, 9.15)	Visi 5ug/kg	
Clinical Remission															

Relative risk with 95% confidence intervals in parentheses. Comparisons between columns and rows should be read from left to right. The blue boxes represent statistically significant comparisons and the white boxes represent non-statistically significant comparisons.

Note:ADA, adalimumab; ETR, etrolizumab; GOL, golimumab; IFX, infliximab; UST, ustekinumab; VED, vedolizumab; MIR, mirikizumab; Visi, visilizumab.

When evaluating maintenance of clinical remission, 17 RCTs provided data for this endpoint (Sandborn et al., 2012; Suzuki et al., 2014; Sandborn et al., 2020; Peyrin-Biroulet et al., 2022; Danese et al., 2022; Panés et al., 2022; Rutgeerts et al., 2005; Jiang et al., 2015; D'Haens et al., 2023; Sandborn et al., 2014b; Sands et al., 2019; Feagan et al., 2013; Motoya et al., 2019; Sands et al., 2019b; Hibi et al., 2017; Vermeire et al., 2022). Except for etrolizumab 105 mg, golimumab 50 mg, infliximab 3.5 mg/kg, and ustekinumab 90 mg every 12 weeks, all interventions were superior to placebo based on direct meta-analysis with low heterogeneity (I^2^ = 0%) (Figure 1B). Vedolizumab 300 mg every 8 weeks was superior to etrolizumab 105 mg (RR 1.79; 95% CI 1.02–3.13) (Table 2). For ranking by SUCRA, vedolizumab 108 mg every other week (SUCRA 0.820), vedolizumab 300 mg every 4 weeks (SUCRA 0.792) and adalimumab 40 mg every other week (SUCRA 0.788) ranked the highest for the maintenance of clinical remission (Supplementary Figure S6).

**TABLE 2:** League table for achieving clinical remission and endoscopic improvement of the maintenance phase: all patients.

ADA 40 mg EOW	1.25 (0.89, 1.77)	1 (0.67, 1.5)	1.13 (0.73, 1.82)	1.04 (0.65, 1.71)	1.32 (0.89, 1.97)	1.19 (0.68, 2.1)	1.18 (0.83, 1.73)	1.07 (0.7, 1.63)	0.6 (0.45, 0.78)	0.9 (0.57, 1.46)	1.06 (0.67, 1.7)	1.52 (0.98, 2.35)	1.57 (1.06, 2.35)	1.42 (1.08, 1.84)	Endoscopic Improvement
0.73 (0.4, 1.31)	ADA 40 mg QW	0.8 (0.48, 1.37)	0.9 (0.52, 1.64)	0.83 (0.46, 1.54)	1.06 (0.63, 1.79)	0.95 (0.49, 1.86)	0.95 (0.58, 1.59)	0.86 (0.5, 1.48)	0.48 (0.31, 0.74)	0.73 (0.41, 1.31)	0.86 (0.48, 1.52)	1.22 (0.7, 2.12)	1.26 (0.75, 2.14)	1.14 (0.73, 1.75)	
1.43 (0.82, 2.68)	1.95 (0.89, 4.73)	ETR 105 mg	1.12 (0.72, 1.82)	1.04 (0.63, 1.72)	1.31 (0.92, 1.88)	1.18 (0.69, 2.01)	1.18 (0.88, 1.59)	1.06 (0.69, 1.64)	0.59 (0.44, 0.79)	0.9 (0.55, 1.47)	1.06 (0.66, 1.71)	1.51 (0.92, 2.5)	1.57 (0.98, 2.52)	1.41 (0.95, 2.08)	
1.02 (0.47, 1.88)	1.39 (0.52, 3.19)	0.71 (0.32, 1.3)	GOL 100 mg	0.93 (0.63, 1.3)	1.17 (0.72, 1.8)	1.05 (0.55, 1.9)	1.05 (0.67, 1.6)	0.95 (0.57, 1.49)	0.53 (0.36, 0.74)	0.8 (0.46, 1.34)	0.95 (0.54, 1.56)	1.34 (0.77, 2.27)	1.39 (0.82, 2.29)	1.25 (0.78, 1.9)	
1.17 (0.55, 2.44)	1.6 (0.62, 4.11)	0.82 (0.37, 1.64)	1.14 (0.69, 2.21)	GOL 50 mg	1.26 (0.76, 2.05)	1.14 (0.59, 2.15)	1.13 (0.7, 1.82)	1.03 (0.61, 1.69)	0.57 (0.38, 0.84)	0.87 (0.49, 1.52)	1.02 (0.59, 1.76)	1.46 (0.82, 2.57)	1.51 (0.87, 2.57)	1.36 (0.83, 2.16)	
0.86 (0.47, 1.62)	1.17 (0.51, 2.81)	0.6 (0.34, 1.03)	0.84 (0.45, 1.89)	0.73 (0.35, 1.61)	INF 10 mg/kg	0.9 (0.54, 1.51)	0.9 (0.7, 1.16)	0.81 (0.53, 1.24)	0.45 (0.34, 0.6)	0.69 (0.42, 1.11)	0.81 (0.5, 1.29)	1.15 (0.7, 1.89)	1.19 (0.74, 1.91)	1.07 (0.73, 1.57)	
1.08 (0.51, 2.41)	1.48 (0.58, 4.03)	0.76 (0.36, 1.56)	1.07 (0.49, 2.73)	0.93 (0.39, 2.33)	1.26 (0.62, 2.62)	INF 3.5 mg/kg	1 (0.63, 1.6)	0.9 (0.5, 1.63)	0.5 (0.31, 0.82)	0.77 (0.4, 1.44)	0.9 (0.48, 1.68)	1.28 (0.67, 2.42)	1.33 (0.72, 2.49)	1.19 (0.68, 2.09)	
1.04 (0.6, 1.86)	1.42 (0.65, 3.3)	0.73 (0.46, 1.12)	1.02 (0.57, 2.19)	0.89 (0.45, 1.88)	1.21 (0.8, 1.85)	0.96 (0.51, 1.78)	INF 5 mg/kg	0.9 (0.6, 1.34)	0.5 (0.39, 0.64)	0.77 (0.48, 1.22)	0.9 (0.57, 1.41)	1.28 (0.79, 2.06)	1.33 (0.84, 2.07)	1.2 (0.83, 1.69)	
1.04 (0.53, 2.23)	1.42 (0.59, 3.77)	0.73 (0.36, 1.48)	1.02 (0.52, 2.57)	0.89 (0.4, 2.17)	1.21 (0.6, 2.59)	0.96 (0.41, 2.33)	1 (0.51, 2.02)	MIR 200 mg	0.56 (0.4, 0.76)	0.85 (0.52, 1.41)	1 (0.61, 1.63)	1.42 (0.85, 2.4)	1.47 (0.91, 2.4)	1.33 (0.88, 1.99)	
2.1 (1.42, 3.34)	2.86 (1.46, 6.2)	1.47 (0.97, 2.22)	2.06 (1.31, 4.02)	1.79 (1.02, 3.49)	2.44 (1.58, 3.92)	1.94 (1.02, 3.77)	2.02 (1.41, 2.98)	2.01 (1.13, 3.59)	Placebo	1.52 (1.04, 2.27)	1.79 (1.23, 2.62)	2.55 (1.71, 3.87)	2.64 (1.83, 3.85)	2.38 (1.84, 3.1)	
1.3 (0.65, 2.83)	1.78 (0.73, 4.78)	0.91 (0.44, 1.89)	1.28 (0.63, 3.29)	1.11 (0.49, 2.78)	1.52 (0.73, 3.29)	1.2 (0.5, 2.97)	1.25 (0.63, 2.59)	1.25 (0.54, 2.87)	0.62 (0.34, 1.13)	UST 90mgQ12W	1.17 (0.84, 1.66)	1.68 (0.95, 2.93)	1.74 (1.01, 2.99)	1.57 (0.97, 2.49)	
1.14 (0.57, 2.46)	1.56 (0.64, 4.19)	0.8 (0.39, 1.65)	1.12 (0.56, 2.86)	0.98 (0.43, 2.41)	1.33 (0.64, 2.86)	1.05 (0.44, 2.58)	1.1 (0.55, 2.24)	1.1 (0.48, 2.51)	0.54 (0.3, 0.99)	0.88 (0.49, 1.55)	UST 90mgQ8W	1.43 (0.82, 2.48)	1.48 (0.87, 2.53)	1.33 (0.84, 2.11)	
0.7 (0.36, 1.43)	0.96 (0.4, 2.45)	0.49 (0.23, 1.02)	0.69 (0.33, 1.74)	0.6 (0.26, 1.47)	0.82 (0.38, 1.77)	0.65 (0.26, 1.59)	0.68 (0.33, 1.39)	0.68 (0.28, 1.54)	0.34 (0.18, 0.61)	0.54 (0.22, 1.26)	0.61 (0.26, 1.44)	VED 108mgEOW	1.04 (0.64, 1.67)	0.93 (0.64, 1.34)	
0.73 (0.4, 1.48)	1.01 (0.44, 2.57)	0.52 (0.26, 1.05)	0.72 (0.37, 1.8)	0.63 (0.29, 1.54)	0.86 (0.42, 1.83)	0.68 (0.29, 1.65)	0.71 (0.37, 1.44)	0.71 (0.32, 1.59)	0.35 (0.2, 0.63)	0.57 (0.25, 1.3)	0.65 (0.28, 1.48)	1.05 (0.5, 2.33)	VED 300mgQ4W	0.9 (0.64, 1.24)	
0.79 (0.54, 1.29)	1.09 (0.55, 2.38)	0.56 (0.32, 0.98)	0.78 (0.44, 1.71)	0.68 (0.35, 1.47)	0.93 (0.53, 1.7)	0.74 (0.35, 1.58)	0.77 (0.46, 1.32)	0.76 (0.39, 1.52)	0.38 (0.26, 0.55)	0.61 (0.3, 1.24)	0.7 (0.35, 1.41)	1.13 (0.65, 2.07)	1.08 (0.63, 1.86)	VED 300mgQ8W	
Clinical Remission															

Relative risk with 95% confidence intervals in parentheses. Comparisons between columns and rows should be read from left to right. The blue boxes represent statistically significant comparisons and the white boxes represent non-statistically significant comparisons.

ADA, adalimumab; ETR, etrolizumab; GOL, golimumab; IFX, infliximab; UST, ustekinumab; VED, vedolizumab; MIR, mirikizumab.

EOW, every other week; QW, every week; Q12W, every 12 weeks; Q8W, every 8 weeks; Q4W, every 4 weeks.

14 trials reported induction of clinical remission in the subset of biologic-naive patients with low heterogeneity (I^2^ = 0%) (Reinisch et al., 2011; Sandborn et al., 2012; Suzuki et al., 2014; Rubin et al., 2022; Danese et al., 2022; Rutgeerts et al., 2005; Jiang et al., 2015; D'Haens et al., 2023; Sandborn et al., 2014a; Sands et al., 2019; Motoya et al., 2019; Sands et al., 2019). Other than ustekinumab 6 mg/kg and adalimumab 80/40 mg, other biologics were superior to placebo in direct comparison (Figure 1C). In indirect comparison, infliximab 5 mg/kg was superior to adalimumab 80/40 mg (RR 1.53; 95% CI 1.01–2.41), adalimumab 160/80 mg (RR 2.25; 95% CI 1.22–4.37) and etrolizumab 105 mg (RR 1.72; 95% CI 1.16–2.60) (Supplementary Table S3). Infliximab 5 mg/kg (SUCRA 0.841) ranked first for this endpoint, with golimumab 400/200 mg s (SUCRA 0.738), golimumab 200/100 mg third (SUCRA 0.737) and infliximab 3.5 mg/kg fourth (SUCRA 0.712) (Supplementary Figure S7).

Six RCTs evaluated the induction phase of clinical remission in a total of 2,264 biologic-exposed patients with low heterogeneity (I^2^ = 0%) (Sandborn et al., 2012; Peyrin-Biroulet et al., 2022; D'Haens et al., 2023; Sands et al., 2019; Motoya et al., 2019; Sands et al., 2019). No significant differences were observed between biological drugs and placebo for this outcome (Supplementary Table S3; Figure 1D). As for the rank by SUCRA values, ustekinumab 6 mg/kg (SUCRA 0.796) ranked first, while adalimumab 160/80 mg last (0.224) (Supplementary Figure S8).

### Clinical response

**FIGURE 2:**
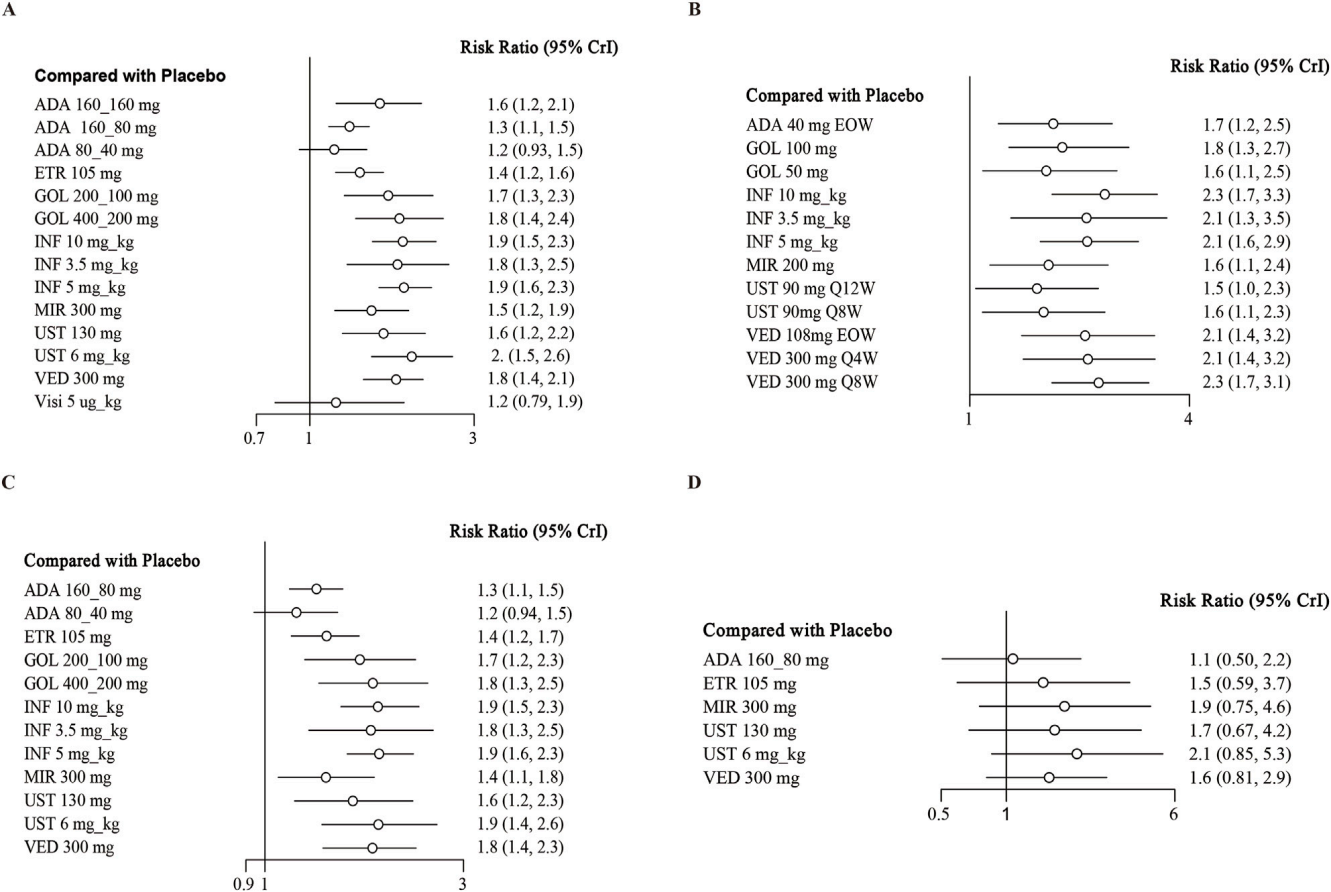
Forest plot for achieving clinical response in **(A)** induction therapy: all patients, **(B)** maintenance therapy: all patients, **(C)** biologic-naive patients, **(D)** biologic-exposed patients. Note: ADA, adalimumab; ETR, etrolizumab; GOL, golimumab; IFX, infliximab; UST, ustekinumab; VED, vedolizumab; MIR, mirikizumab; Visi, visilizumab; EOW, every other week; QW, every week; Q12W, every 12 weeks; Q8W, every 8 weeks; Q4W, every 4 weeks.

When assessing the induction of clinical response, there were 18 trials evaluating this endpoint and showed low heterogeneity (I^2^ = 0%) (Higgins et al., 2012; Reinisch et al., 2011; Sandborn et al., 2012; Sandborn et al., 2010; Suzuki et al., 2014; Peyrin-Biroulet et al., 2022; Rubin et al., 2022; Danese et al., 2022; Panés et al., 2022; Rutgeerts et al., 2005; Jiang et al., 2015; D'Haens et al., 2023; Sandborn et al., 2014a; Sands et al., 2019a; Feagan et al., 2013; Motoya et al., 2019; Sands et al., 2019b). Except for adalimumab 80/40 mg (RR 1.2; 95% CI, 0.93–1.5) and visilizumab 5 μg/kg (RR 1.2; 95% CI, 0.79–1.9), other interventions were superior to placebo (Figure 2A). In indirect comparison, infliximab 10 mg/kg, infliximab 5 mg/kg and ustekinumab 6 mg/kg were superior to adalimumab 160/80 mg and etrolizumab 105 mg (Table 3). The ranking of probability by SUCRA indicated that ustekinumab 6 mg/kg ranked first (SUCRA 0.849), followed by infliximab 5 mg/kg (SUCRA 0.791), infliximab 10 mg/kg third (SUCRA 0.771), and golimumab 400/200 mg forth (SUCRA 0.729) (Supplementary Figure S5).

**TABLE 3:** League table for achieving clinical response of the induction phase: all patients.

ADA 160/160 mg														
1.22 (0.95, 1.57)	ADA 160/80 mg													
1.36 (0.97, 1.89)	1.11 (0.89, 1.39)	ADA 80/40 mg												
1.14 (0.84, 1.54)	0.93 (0.79, 1.1)	0.84 (0.65, 1.08)	ETR 105 mg											
0.95 (0.62, 1.41)	0.77 (0.56, 1.06)	0.7 (0.48, 1)	0.83 (0.59, 1.15)	GOL 200/100 mg										
0.88 (0.58, 1.31)	0.72 (0.52, 0.99)	0.65 (0.45, 0.93)	0.77 (0.55, 1.07)	0.93 (0.72, 1.2)	GOL 400/200 mg									
0.86 (0.59, 1.19)	0.7 (0.54, 0.88)	0.63 (0.46, 0.84)	0.75 (0.58, 0.94)	0.91 (0.63, 1.3)	0.97 (0.67, 1.39)	INF 10 mg/kg								
0.89 (0.57, 1.36)	0.72 (0.5, 1.03)	0.65 (0.44, 0.97)	0.78 (0.54, 1.1)	0.94 (0.6, 1.47)	1.02 (0.64, 1.57)	1.04 (0.73, 1.49)	INF 3.5 mg/kg							
0.85 (0.6, 1.16)	0.69 (0.56, 0.84)	0.63 (0.47, 0.82)	0.74 (0.61, 0.89)	0.9 (0.63, 1.26)	0.97 (0.68, 1.35)	0.99 (0.83, 1.19)	0.96 (0.7, 1.31)	INF 5 mg/kg						
1.06 (0.72, 1.53)	0.86 (0.65, 1.14)	0.78 (0.55, 1.08)	0.92 (0.69, 1.23)	1.12 (0.76, 1.65)	1.2 (0.82, 1.76)	1.23 (0.9, 1.72)	1.19 (0.79, 1.82)	1.24 (0.93, 1.7)	MIR 300 mg					
1.6 (1.19, 2.11)	1.3 (1.14, 1.49)	1.18 (0.93, 1.46)	1.4 (1.19, 1.64)	1.69 (1.26, 2.28)	1.82 (1.36, 2.44)	1.86 (1.52, 2.33)	1.8 (1.28, 2.53)	1.88 (1.59, 2.26)	1.51 (1.18, 1.94)	Placebo				
0.98 (0.65, 1.44)	0.8 (0.58, 1.08)	0.72 (0.5, 1.02)	0.85 (0.62, 1.17)	1.03 (0.69, 1.55)	1.11 (0.74, 1.66)	1.14 (0.81, 1.62)	1.1 (0.71, 1.71)	1.15 (0.83, 1.6)	0.92 (0.64, 1.34)	0.61 (0.46, 0.8)	UST 130 mg			
0.81 (0.54, 1.19)	0.66 (0.49, 0.89)	0.6 (0.41, 0.84)	0.71 (0.51, 0.96)	0.86 (0.57, 1.27)	0.92 (0.62, 1.37)	0.94 (0.68, 1.34)	0.91 (0.6, 1.41)	0.95 (0.69, 1.32)	0.76 (0.53, 1.1)	0.51 (0.39, 0.66)	0.83 (0.65, 1.06)	UST 6 mg/kg		
0.9 (0.67, 1.26)	0.73 (0.62, 0.9)	0.66 (0.51, 0.89)	0.78 (0.63, 1.01)	0.95 (0.68, 1.39)	1.02 (0.73, 1.49)	1.05 (0.8, 1.44)	1.01 (0.69, 1.53)	1.05 (0.83, 1.41)	0.85 (0.63, 1.19)	0.56 (0.47, 0.7)	0.92 (0.67, 1.33)	1.11 (0.81, 1.59)	VED 300 mg	
1.34 (0.77, 2.19)	1.09 (0.68, 1.67)	0.98 (0.59, 1.57)	1.17 (0.72, 1.82)	1.42 (0.83, 2.34)	1.52 (0.89, 2.51)	1.56 (0.95, 2.5)	1.51 (0.85, 2.58)	1.57 (0.97, 2.47)	1.27 (0.75, 2.04)	0.84 (0.53, 1.26)	1.37 (0.81, 2.24)	1.66 (0.97, 2.69)	1.49 (0.89, 2.32)	Visi 5ug/kg

Relative risk with 95% confidence intervals in parentheses. Comparisons between columns and rows should be read from left to right. The blue boxes represent statistically significant comparisons and the white boxes represent non-statistically significant comparisons.

ADA, adalimumab; ETR, etrolizumab; GOL, golimumab; IFX, infliximab; UST, ustekinumab; VED, vedolizumab; MIR, mirikizumab;Visi, visilizumab.

Regarding the maintenance of clinical response, 12 independent RCTs from 11 studies reported this outcome, involving a total of 3913 patients (Sandborn et al., 2012; Suzuki et al., 2014; Sandborn et al., 2020; Rutgeerts et al., 2005; Jiang et al., 2015; D'Haens et al., 2023; Sandborn et al., 2014a; Sands et al., 2019b; Feagan et al., 2013; Motoya et al., 2019; Hibi et al., 2017). When we pooled the data, all interventions were found to be superior to placebo in direct comparisons (Figure 2B). In network meta-analysis comparing active treatments, no significant differences were observed between the interventions (Table 4). Vedolizumab 108 mg every other week ranked first (SUCRA 0.820), followed by vedolizumab 300 mg every 4 weeks (SUCRA 0.792), adalimumab 40 mg once a week (SUCRA 0.788), and vedolizumab 300 mg every 8 weeks (SUCRA 0.747) for the maintenance of clinical response (Supplementary Figure S6).

**TABLE 4:** League table for achieving clinical response of the maintenance phase: all patients.

ADA 40 mg EOW												
0.94 (0.54, 1.54)	GOL 100 mg											
1.05 (0.59, 1.79)	1.1 (0.77, 1.69)	GOL 50 mg										
0.72 (0.45, 1.19)	0.77 (0.48, 1.31)	0.69 (0.41, 1.21)	INF 10 mg/kg									
0.81 (0.44, 1.48)	0.86 (0.47, 1.62)	0.78 (0.41, 1.48)	1.12 (0.66, 1.84)	INF 3.5 mg/kg								
0.81 (0.5, 1.29)	0.85 (0.54, 1.42)	0.77 (0.46, 1.31)	1.12 (0.83, 1.47)	0.99 (0.64, 1.55)	INF 5 mg/kg							
1.03 (0.62, 1.73)	1.09 (0.68, 1.94)	0.98 (0.57, 1.77)	1.43 (0.87, 2.34)	1.27 (0.7, 2.37)	1.28 (0.81, 2.09)	MIR 200 mg						
1.69 (1.2, 2.46)	1.79 (1.28, 2.73)	1.62 (1.08, 2.53)	2.34 (1.68, 3.27)	2.09 (1.3, 3.47)	2.1 (1.56, 2.9)	1.64 (1.13, 2.39)	Placebo					
1.11 (0.66, 1.9)	1.17 (0.72, 2.11)	1.06 (0.61, 1.93)	1.54 (0.92, 2.56)	1.37 (0.74, 2.61)	1.38 (0.85, 2.28)	1.08 (0.63, 1.84)	0.65 (0.44, 0.96)	UST 90 mg Q12W				
1.06 (0.63, 1.82)	1.12 (0.69, 2.02)	1.02 (0.59, 1.84)	1.47 (0.88, 2.45)	1.31 (0.71, 2.5)	1.32 (0.81, 2.19)	1.03 (0.6, 1.76)	0.63 (0.43, 0.92)	0.96 (0.66, 1.38)	UST 90 mg Q8W			
0.82 (0.47, 1.41)	0.86 (0.51, 1.57)	0.78 (0.44, 1.43)	1.13 (0.66, 1.91)	1.01 (0.53, 1.94)	1.01 (0.6, 1.7)	0.79 (0.45, 1.37)	0.48 (0.31, 0.72)	0.74 (0.41, 1.29)	0.77 (0.43, 1.35)	VED 108 mg EOW		
0.8 (0.47, 1.4)	0.85 (0.51, 1.55)	0.77 (0.43, 1.41)	1.11 (0.65, 1.89)	0.99 (0.53, 1.92)	1 (0.6, 1.68)	0.78 (0.45, 1.35)	0.47 (0.31, 0.71)	0.73 (0.41, 1.28)	0.76 (0.42, 1.33)	0.98 (0.59, 1.66)	VED 300 mg Q4W	
0.75 (0.47, 1.2)	0.79 (0.51, 1.34)	0.72 (0.43, 1.23)	1.04 (0.66, 1.63)	0.92 (0.53, 1.69)	0.93 (0.61, 1.45)	0.73 (0.45, 1.17)	0.44 (0.32, 0.6)	0.68 (0.41, 1.1)	0.71 (0.43, 1.15)	0.92 (0.64, 1.36)	0.93 (0.64, 1.37)	VED 300 mg Q8W

Relative risk with 95% confidence intervals in parentheses. Comparisons between columns and rows should be read from center to right. The blue boxes represent statistically significant comparisons and the white boxes represent non-statistically significant comparisons.

ADA, adalimumab; ETR, etrolizumab; GOL, golimumab; IFX, infliximab; UST, ustekinumab; VED, vedolizumab; MIR, mirikizumab;Visi, visilizumab.

14 trials reported the induction of clinical response in a subset of biologics-naive patients (Reinisch et al., 2011; Sandborn et al., 2012; Suzuki et al., 2014; Rubin et al., 2022; Danese et al., 2022; Rutgeerts et al., 2005; Jiang et al., 2015; D'Haens et al., 2023; Sandborn et al., 2014a; Sands et al., 2019; Motoya et al., 2019; Sands et al., 2019), recruiting 5538 patients. When data were pooled, other than adalimumab 80/40 mg, all drugs were superior to placebo in the induction of clinical response with low heterogeneity (I^2^ = 0%) (Figure 2C). In indirect comparison, aside from the results of direct comparisons, vedolizumab 300 mg was superior to adalimumab 160/80 mg (RR 1.36; 95% CI 1.07–1.69) (Supplementary Table S4). Other significantly different comparison among biologics for this outcome were summarized in Supplementary Table S4. For this endpoint, infliximab 5 mg/kg (SUCRA 0.777) ranked first, followed by infliximab 10 mg/kg second (SUCRA 0.761), ustekinumab 6 mg/kg (SUCRA 0.760) and golimumab 400/200 mg (SUCRA 0.714) (Supplementary Figure S7).

Finally, seven RCTs reported on the induction of clinical response in the subset of biologic-exposed patients (Sandborn et al., 2012; Peyrin-Biroulet et al., 2022; D'Haens et al., 2023; Sands et al., 2019; Feagan et al., 2013; Motoya et al., 2019; Sands et al., 2019). There were 2129 patients included in these trials, and low heterogeneity among them (I^2^ = 36.88%). None significant difference between biologics and placebo were observed for this outcome (Supplementary Table S4; Figure 2D). As for the rank among biologics, ustekinumab 6 mg/kg (SUCRA 0.796), mirikizumab 300 mg (0.786) and ustekinumab 130 mg/kg (0.588) ranked the highest in this endpoint (Supplementary Figure S8).

### Endoscopic improvement

**FIGURE 3:**
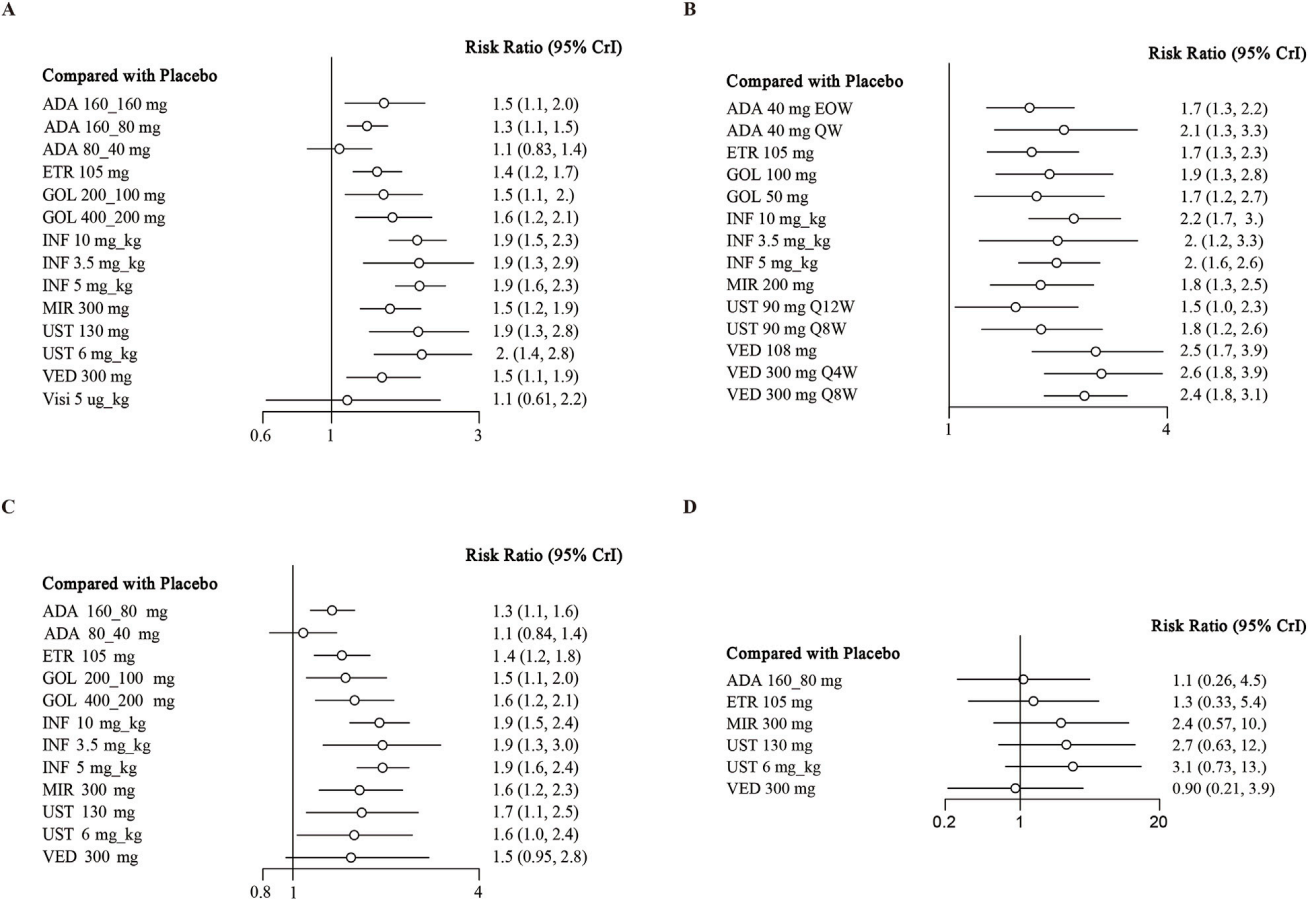
Forest plot for achieving endoscopic improvement in **(A)** induction therapy: all patients, **(B)** maintenance therapy: all patients, **(C)** biologic-naive patients, **(D)** biologic-exposed patients. Note: ADA, adalimumab; ETR, etrolizumab; GOL, golimumab; IFX, infliximab; UST, ustekinumab; VED, vedolizumab; MIR, mirikizumab; Visi, visilizumab; EOW, every other week; QW, every week; Q12W, every 12 weeks; Q8W, every 8 weeks; Q4W, every 4 weeks.

When evaluating the induction therapy of endoscopic improvement, in total, 17 RCTs reported data for this endpoint including 8183 patients (Reinisch et al., 2011; Sandborn et al., 2012; Sandborn et al., 2010; Suzuki et al., 2014; Peyrin-Biroulet et al., 2022; Rubin et al., 2022; Danese et al., 2022; Panés et al., 2022; Rutgeerts et al., 2005; Jiang et al., 2015; D'Haens et al., 2023; Sandborn et al., 2014a; Sands et al., 2019; Feagan et al., 2013; Motoya et al., 2019). Except for adalimumab 80/40 mg and visilizumab 5 μg/kg, other biologics were superior to placebo in direct comparison for this endpoint (Figure 3A). When comparing active treatments, ustekinumab 6 mg/kg was superior to adalimumab 160/80 mg (RR 0.66 95% CI, 0.45–0.98), while infliximab 5 mg/kg and 10 mg/kg were superior to adalimumab 160/80 mg and etrolizumab 105 mg (Table 1). The SUCRA values indicated that infliximab 5 mg/kg (SUCRA 0.834) ranked first, ustekinumab 6 mg/kg ranked second (SUCRA 0.812), followed by infliximab 10 mg/kg (SUCRA 0.805) (Supplementary Figure S5).

When evaluating the maintenance therapy of endoscopic improvement, 17 trials reported data for this endpoint including 5859 patients (Sandborn et al., 2012; Suzuki et al., 2014; Sandborn et al., 2020; Peyrin-Biroulet et al., 2022; Danese et al., 2022; Panés et al., 2022; Rutgeerts et al., 2005; Jiang et al., 2015; D'Haens et al., 2023; Sandborn et al., 2014a; Sands et al., 2019a; Feagan et al., 2013; Motoya et al., 2019; Sands et al., 2019b; Hibi et al., 2017; Vermeire et al., 2022). All interventions were superior to placebo on direct meta-analysis with low heterogeneity (I^2^ = 0%) (Figure 3B). In network meta-analysis, vedolizumab 300 mg every 8 weeks and vedolizumab 300 mg every 4 weeks were superior to adalimumab 40 mg every other week (Table 2). Vedolizumab 300 mg every 12 weeks (SUCRA 0.880), vedolizumab 108 mg every other week (SUCRA 0.835) and vedolizumab 300 mg every 8 weeks (SUCRA 0.785) ranked the highest for this endpoint (Supplementary Figure S6).

There are 13 trials from 11 studies reported the induction therapy of endoscopic improvement in a subset of biologic-naive patients (Reinisch et al., 2011; Sandborn et al., 2012; Suzuki et al., 2014; Rubin et al., 2022; Danese et al., 2022; Rutgeerts et al., 2005; Jiang et al., 2015; D'Haens et al., 2023; Sandborn et al., 2014; Sands et al., 2019; Motoya et al., 2019), recruiting 4929 patients with low heterogeneity (I^2^ = 0). Except for adalimumab 80/40 mg and vedolizumab 300 mg, other interventions were superior to placebo (Figure 3C). On meta-analysis, infliximab 5 mg/kg ranked first (SUCRA 0.841), followed by infliximab 10 mg/kg (SUCRA 0.801) (Supplementary Figure S7). Adalimumab 80/40 mg was inferior to infliximab 3.5 mg/kg (RR 0.56, 95% CI, 0.34–0.91) and mirikizumab 300 mg (RR 0.66, 95% CI, 0.44–0.97) (Supplementary Table S5).

Finally, 4 RCTs reported on endoscopic improvement in a subset of biologic-exposed patients (Sandborn et al., 2012; D'Haens et al., 2023; Sands et al., 2019; Motoya et al., 2019), and one trials only recruited them (Suzuki et al., 2014). A total of 1801 patients included in these five RCTs. However, no significant difference was observed between biologics and placebo (Supplementary Table S5; Figure 3D). And the rank of SUCRA values was exhibited in Supplementary Figure S8.

### Adverse events

In the induction phase, 11 RCTs reported the total number of adverse events from 6601 patients with low heterogeneity (I^2^ = 0%) (Reinisch et al., 2011; Sandborn et al., 2010; Suzuki et al., 2014; Peyrin-Biroulet et al., 2022; Rubin et al., 2022; Panés et al., 2022; D'Haens et al., 2023; Sandborn et al., 2014a; Sands et al., 2019; Motoya et al., 2019). When data were pooled, visilizumab 5 μg/kg was more likely to lead to adverse events than placebo (RR 0.79, 95% CI, 0.6–0.99) (Supplementary Figure 9A; Supplementary Table S6). In indirect comparison, visilizumab 5 μg/kg was more likely to lead to adverse events than ustekinumab 130 mg (RR 0.67, 95% CI, 0.47–0.95) (Supplementary Table S6). As for the rankings, ustekinumab 130 mg (SUCRA 0.869) and mirikizumab 300 mg (SUCRA 0.638) were the safest drugs (Supplementary Figure S10). As for the serious adverse events in induction phase, network meta-analysis revealed no significant difference between active treatments in 11 RCTs including 6601 patients (Reinisch et al., 2011; Sandborn et al., 2010; Suzuki et al., 2014; Peyrin-Biroulet et al., 2022; Rubin et al., 2022; Panés et al., 2022; D'Haens et al., 2023; Sandborn et al., 2014a; Sands et al., 2019; Motoya et al., 2019). No significant differences were observed between biologics and placebo (Supplementary Figure S9B; Supplementary Table S6). The safety profile of golimumab 200/100 mg ranked first (SUCRA = 0.737), whereas etrolizumab 105 mg had the lowest safety (SUCRA = 0.169) (Supplementary Figure 10). In terms of infections in induction therapy, the data from 10 RCTs were pooled (Reinisch et al., 2011; Sandborn et al., 2010; Suzuki et al., 2014; Peyrin-Biroulet et al., 2022; Rubin et al., 2022; Panés et al., 2022; D'Haens et al., 2023; Sandborn et al., 2014a; Sands et al., 2019a), there was no significant differences were observed between biologics and placebo (Supplementary Figure S9C; Supplementary Table S7). As for the rank, adalimumab 160/160 mg ranked first (SUCRA 0.715), while visilizumab 5 μg/kg ranked last (SUCRA 0.334) (Supplementary Figure S10). Finally, 9 RCTs reported adverse events leading to the discontinuation of study drugs among 5514 patients (Reinisch et al., 2011; Suzuki et al., 2014; Peyrin-Biroulet et al., 2022; Rubin et al., 2022; Panés et al., 2022; D'Haens et al., 2023; Sandborn et al., 2014a; Motoya et al., 2019). However, no significant difference was observed in the adverse events leading to withdrawal between the various biologic agents or compared with placebo (Supplementary Figure S9D; Supplementary Table S7), and the ranks were showed in Supplementary Figure S10.

When evaluating safety in the maintenance phase, in terms of the total number of adverse events, 16 RCTs reported data from 6490 patients with low heterogeneity (I^2^ = 6%) (Sandborn et al., 2012; Sandborn et al., 2020; Peyrin-Biroulet et al., 2022; Danese et al., 2022; Panés et al., 2022; Rutgeerts et al., 2005; Jiang et al., 2015; D'Haens et al., 2023; Sandborn et al., 2014b; Sands et al., 2019a; Feagan et al., 2013; Motoya et al., 2019; Sands et al., 2019b; Hibi et al., 2017; Vermeire et al., 2022). Vedolizumab 108 mg (SUCRA 0.869) and ustekinumab 90 mg every 12 weeks (SUCRA 0.850) were the safest drugs. And golimumab 100 mg was the most likely drug to cause adverse events (SUCRA 0.092) (Supplementary Figure S11). Golimumab 100 mg was more likely to lead to adverse events than placebo (RR 1.19, 95% CI, 1.02–1.42) and ustekinumab 90 mg every 12 weeks (RR 1.36, 95% CI, 1.06–1.78) as well as vedolizumab 108 mg (RR 1.4, 95% CI, 1.06–1.86) (Supplementary Figure S12A; Supplementary Table S8). For the serious adverse events, there did not show statistically significant difference between biologics and placebo in 16 trials including 6, 490 patients (Supplementary Figure S12B) (Sandborn et al., 2012; Sandborn et al., 2020; Peyrin-Biroulet et al., 2022; Danese et al., 2022; Panés et al., 2022; Rutgeerts et al., 2005; Jiang et al., 2015; D'Haens et al., 2023; Sandborn et al., 2014b; Sands et al., 2019a; Feagan et al., 2013; Motoya et al., 2019; Sands et al., 2019b; Hibi et al., 2017; Vermeire et al., 2022). In network meta-analysis, mirikizumab 200 mg ranked first (SUCRA 0.882), while golimumab 100 mg ranked last (SUCRA 0.189) (Supplementary Figure S11). Etrolizumab 105 mg was more likely to cause serious adverse events than mirikizumab 200 mg (RR 2.98, 95% CI, 1.05–8.89) (Supplementary Table S8). In terms of infections, when we pooled the data from 13 RCTs, recruiting 5515 patients (Sandborn et al., 2012; Sandborn et al., 2020; Peyrin-Biroulet et al., 2022; Danese et al., 2022; Panés et al., 2022; Rutgeerts et al., 2005; Jiang et al., 2015; D'Haens et al., 2023; Sandborn et al., 2014b; Sands et al., 2019a; Feagan et al., 2013; Hibi et al., 2017; Vermeire et al., 2022). In direct comparison, golimumab 50 mg and golimumab 100 mg were more likely to lead to infections than placebo (Supplementary Figure S12C). In indirect comparison, ustekinumab 90 mg every 12 weeks was safer than etrolizumab 105 mg (RR 1.62, 95% CI, 1.05–2.47) and infliximab 10 mg/kg (RR 1.68, 95% CI, 1.07–2.62) (Supplementary Table S9) and the ranks were exhibited in Supplementary Figure S11. Finally, when assessing withdrawals due to adverse events from 13 RCTs (Sandborn et al., 2012; Sandborn et al., 2020; Peyrin-Biroulet et al., 2022; Danese et al., 2022; Panés et al., 2022; Rutgeerts et al., 2005; Jiang et al., 2015; D'Haens et al., 2023; Sandborn et al., 2014b; Sands et al., 2019a; Feagan et al., 2013; Hibi et al., 2017; Vermeire et al., 2022), mirikizumab 200 mg was significantly safer than placebo (RR 0.08, 95% CI, 0.04–0.8) (Supplementary Figure S12D; Supplementary Table S9). Among included biologics, mirikizumab 200 mg ranked first (SUCRA 0.868), followed by ustekinumab 90 mg every 12 weeks (SUCRA 0.815), while golimumab 100 mg ranked last (SUCRA 0.132) (Supplementary Figure S11). Golimumab 100 mg was more likely to lead to discontinuation than mirikizumab 200 mg (RR 8.05, 95% CI, 1.02–65.92) (Supplementary Table S9).

## Discussion

In this updated systematic review and network meta-analysis, we evaluated the efficacy and safety of biologics for moderate to severe UC. This study evaluated infliximab, adalimumab, vedolizumab, mirikizumab, golimumab, ustekinumab, visilizumab, and etrolizumab regarding the induction and maintenance of clinical remission, endoscopic improvement, clinical response, and safety from 23 RCTs across 20 studies, which collectively recruited 10,839 patients.

In induction therapy, all eligible biologics demonstrated significantly greater efficacy compared to placebo, except for adalimumab 80/40 mg and visilizumab 5 μg/kg. Regarding clinical remission and endoscopic improvement, infliximab 5 mg/kg ranked first among all biologics, while ustekinumab 6 mg/kg showed the most effective in achieving clinical response, followed by infliximab 5 mg/kg. In maintenance therapy, except for ustekinumab 90 mg every 12 weeks and etrolizumab 105 mg every 4 weeks, others were significantly better than placebo in achieving clinical remission. In terms of clinical response and endoscopic improvement in maintenance phase, vedolizumab 108 mg every other week and vedolizumab 300 mg every 4 weeks exerted their excellent efficacy. Meanwhile, infliximab 10 mg/kg ranked first for clinical response.

Overall, during induction therapy, considering the safety of total number of adverse events, ustekinumab 130 mg demonstrated the most favorable safety profile, while visilizumab 5 μg/kg exhibited the highest risk. None of the drugs were more likely to lead to serious adverse events than placebo, whereas etrolizumab 105 mg was more likely to cause serious adverse events than golimumab 200/100 mg and ustekinumab 6 mg/kg. Regarding infections, adalimumab 160/160 mg was the safest drug, and visilizumab 5 μg/kg exhibited the worst safety profile. As for discontinuation due to adverse events, a novel drug mirikizumab 300 mg, which has recently completed phase Ⅲ clinical trials, demonstrates superior safety.

Significant differences in the safety assessments were also observed in the maintenance therapy. As for total number of adverse events, vedolizumab 108 mg was the safest agent, while golimumab 100 mg ranked last. Regarding serious adverse events, mirikizumab 200 mg and etrolizumab 105 mg ranked the highest and lowest safety, respectively. Considering the safety of infections, ustekinumab 90 mg every 12 weeks performed best. Finally, for discontinuation due to adverse events, mirikizumab 200 mg, ustekinumab 90 mg every 12 weeks and vedolizumab 108 mg ranked first to third. Notably, the clinical trial of visilizumab was discontinued prematurely due to significant safety concerns (Sandborn et al., 2010).

This study identified infliximab 5 mg/kg, ustekinumab 130 mg, and ustekinumab 6 mg/kg as the most efficacious agents for achieving clinical remission during the induction phase. These finding exhibited a slight discrepancy with a recent network meta-analysis, which reported ustekinumab and infliximab as the first- and second-ranked therapies (Shehab et al., 2024). The potential discrepancy may stem from variations in defining clinical remission, as their analysis relied on the PRO-2 score (Shehab et al., 2024). Notably, this study specifically incorporated biologic dosage as an independent variable. Additionally, during the maintenance phase, regimens of vedolizumab 108 mg every other week and vedolizumab 300 mg every 4 weeks demonstrated superior efficacy in clinical remission, aligning with previous reports (Shehab et al., 2024).

Additionally, compared with recent study (Shehab et al., 2024), a subgroup analysis on whether patients had previously used biologics was conducted to further compared the efficacy. Our results demonstrated that vedolizumab exhibited reduced efficacy in patients with prior biologic exposure, a finding consistent with prior observations (Panaccione et al., 2023). A previously published network meta-analysis confirmed infliximab as the most effective agent for inducing clinical remission and endoscopic improvement in biologic-naive patients, while ustekinumab demonstrated the highest therapeutic ranking among biologic-exposed patients (Singh et al., 2024). This study has enhanced this evidence through systematic incorporation of recently updated clinical trial datasets. Furthermore, we performed a separate safety analysis of biologics during the maintenance therapy. The significantly higher infection risk with golimumab highlights the need of rigorous monitoring in maintenance therapy, particularly in elderly or immunocompromised populations.

The divergent efficacy rankings between biologic-naive and biologic-exposed patients highlight the critical need for personalized therapeutic strategies in UC, moving away from a one-size-fits-all approach. Specifically, in biologic-naive populations, infliximab 5 mg/kg demonstrates superior efficacy as the preferred induction agent, while ustekinumab 6 mg/kg may be prioritized for refractory cases due to its robust response in biologic-exposed cohorts. For maintenance therapy, vedolizumab’s favorable safety profile positions it as an optimal choice for patients with comorbidities, whereas mirikizumab offers a promising alternative for those with intolerance to conventional immunosuppression.

This network meta-analysis offers several notable advantages compared to other recent studies. Firstly, it incorporates the most up-to-date evidence, with literature searches extending to May 2024, thereby capturing newly approved agents such as mirikizumab and ustekinumab, which were underrepresented in earlier analyses. This ensures the findings reflect the rapidly evolving therapeutic landscape (D'Haens et al., 2023; Singh et al., 2024; Burr et al., 2021). Secondly, this study employed a Bayesian framework utilizing Markov chain Monte Carlo simulations to directly quantify uncertainty in treatment effects. Unlike traditional frequentist model that solely provide point estimates with confidence intervals, the Bayesian model generates posterior probability distributions, enabling explicit calculation of the probability for each intervention to be ranked as optimal (Burr et al., 2021). This probabilistic quantification offers clinicians more intuitive risk-benefit assessments to inform therapeutic decision-making. Additionally, compared with recent study, a subgroup analysis on whether patients had previously administered biologics, was conducted to further compared the efficacy (Shehab et al., 2024). Fourthly, the exclusion of phase II trials minimizes bias from exploratory studies, strengthening result reliability (Chu et al., 2023). Fifthly, the study comprehensively evaluated both efficacy (clinical remission, clinical response, endoscopic improvement) and safety (total adverse events, serious adverse events, infections, withdrawals due to adverse events), providing a holistic risk-benefit profile (Shehab et al., 2024).

Our study has some limitations, apart from the usual limitations of network meta-analyses. Firstly, this study focused solely on clinical trial data from previously published biologics, excluding grey literature (such as meeting summaries, letters, and other related publications). It may result in some unpublished negative or neutral findings not being included, thereby potentially overestimating the overall effect size. Since grey literature often contains preliminary research or findings that do not reach significance, their inclusion may cause the combined results to shift in an invalid direction. However, considering that grey literature usually lacks complete methodological details or has not undergone strict peer review, its quality may differ from that of published literature, and the impact of these findings still requires careful interpretation. Future studies can further verify the robustness of the current results through systematic retrieval of grey literature. Furthermore, this study exclusively focused on evaluating biologics, whereas the relative efficacy and safety of small molecule drugs, such as upadacitinib, remain unexplored. Future research should broaden its scope to include newer medications and conduct a more comprehensive investigation of their effects. Additionally, the follow-up endpoint times of various studies are inconsistent, which may bring bias to the observation of effectiveness and safety. Finally, given the time span of the included studies, some recent RCTs may have included patients with refractory UC who had failed to other therapies, which could potentially underestimate the overall efficacy, and the heterogeneity in prior use of biologics may confound the assessment of treatment outcomes. Therefore, the lack of significant differences in outcomes among the subset of biologic-exposed patients may also relative to these reasons.

Similar to any indirect comparison, the results of this study should be interpreted with caution. Direct comparisons through head-to-head trials are necessary to fully elucidate the positioning of these therapies. Nonetheless, these findings can assist clinicians in navigating the increasingly complex landscape of therapeutic options for moderate to severe UC and may inform future revisions of evidence-based clinical management guidelines.

Several newer biologics, particularly anti-IL-23 agents such as guselkumab, risankizumab, and brazikumab, are expected to demonstrate positive outcomes in phase Ⅲ clinical trials. Moreover, combination therapy has been shown to be superior to monotherapy in a recent trial (Panaccione et al., 2014), which may have underestimated the efficacy of certain drugs. Therefore, future studies should integrate real-world data to validate long-term safety signals and explore combination therapies (e.g., biologics + small molecule drugs), which may synergistically enhance efficacy beyond monotherapy.

Overall, this systematic review and network meta-analysis demonstrated that infliximab, ustekinumab, and vedolizumab were the most efficacious treatments. Meanwhile, mirikizumab and vedolizumab exhibited superior safety profiles across most outcomes. However, with the paucity of direct comparisons, the reliability of these findings requires further validation through additional clinical trials, real-world studies, and long-term assessments to confirm the overall safety and efficacy of these biologics.

## Data Availability

The original contributions presented in the study are included in the article/Supplementary Material, further inquiries can be directed to the corresponding authors.
