# Supplementary material for: What is the optimal biological therapy for moderate to severe ulcerative colitis: a systematic review and network meta-analysis
Source: Front Pharmacol. 2025 Jul 11;16:1602024. doi: 10.3389/fphar.2025.1602024 (PMC12290297; doi:10.3389/fphar.2025.1602024)
Supplement: Supplementary file 1 [file DataSheet1.docx]

**Supplementary** 1 The searching strategy

("colitis, ulcerative"[MeSH Terms] OR ("colitis ulcerative"[Title/Abstract] OR "idiopathic proctocolitis"[Title/Abstract] OR "ulcerative colitis"[Title/Abstract] OR "colitis gravis"[Title/Abstract] OR "inflammatory bowel disease ulcerative colitis type"[Title/Abstract])) AND (("Infliximab"[MeSH Terms] OR ("Infliximab"[Title/Abstract] OR "mab ca2"[Title/Abstract] OR "monoclonal antibody ca2"[Title/Abstract] OR "Inflectra"[Title/Abstract] OR "Remicade"[Title/Abstract] OR "Renflexis"[Title/Abstract])) OR ("Adalimumab"[MeSH Terms] OR "Adalimumab"[Title/Abstract] OR "Humira"[Title/Abstract] OR "Amjevita"[Title/Abstract] OR "Cyltezo"[Title/Abstract] OR "D2E7"[Title/Abstract]) OR ("Golimumab"[Supplementary Concept] OR ("Golimumab"[Title/Abstract] OR "cnto 148"[Title/Abstract] OR "Simponi"[Title/Abstract])) OR ("Vedolizumab"[Supplementary Concept] OR ("Vedolizumab"[Title/Abstract] OR "Entyvio"[Title/Abstract] OR "MLN0002"[Title/Abstract] OR "MLN02"[Title/Abstract])) OR ("Etrolizumab"[Supplementary Concept] OR ("Etrolizumab"[Title/Abstract] OR "rhumab beta7"[Title/Abstract] OR "anti beta 7"[Title/Abstract] OR "PRO145223"[Title/Abstract])) OR ("Ustekinumab"[MeSH Terms] OR ("Ustekinumab"[Title/Abstract] OR "Stelara"[Title/Abstract] OR "cnto 1275"[Title/Abstract])) OR ("Eldelumab"[Supplementary Concept] OR "Eldelumab"[Title/Abstract] OR "BMS-936557"[Title/Abstract]) OR ("Ontamalimab"[Supplementary Concept] OR ("Ontamalimab"[Title/Abstract] OR ("SHP647"[Title/Abstract] OR "PF-00547659"[Title/Abstract]))) OR ("Basiliximab"[MeSH Terms] OR ("Basiliximab"[Title/Abstract] OR "Simulect"[Title/Abstract] OR "chi 621"[Title/Abstract])) OR ("Visilizumab"[Supplementary Concept] OR ("Visilizumab"[Title/Abstract] OR "Nuvion"[Title/Abstract])) OR ("Guselkumab"[Supplementary Concept] OR ("Guselkumab"[Title/Abstract] OR "Tremfya"[Title/Abstract] OR "cnto 1959"[Title/Abstract])) OR ("Daclizumab"[MeSH Terms] OR ("Daclizumab"[Title/Abstract] OR "Zenapax"[Title/Abstract])) OR ("Mirikizumab"[Supplementary Concept] OR ("Mirikizumab"[Title/Abstract] OR "LY3074828"[Title/Abstract])) OR ("Natalizumab"[MeSH Terms] OR ("Natalizumab"[Title/Abstract] OR "Tysabri"[Title/Abstract] OR "Antegren"[Title/Abstract])) OR ("Certolizumab Pegol"[MeSH Terms] OR ("Certolizumab Pegol"[Title/Abstract] OR "Cimzia"[Title/Abstract] OR "CDP870"[Title/Abstract]))) AND ("randomized controlled trial"[Publication Type] OR "randomized"[Title/Abstract] OR "placebo"[Title/Abstract])


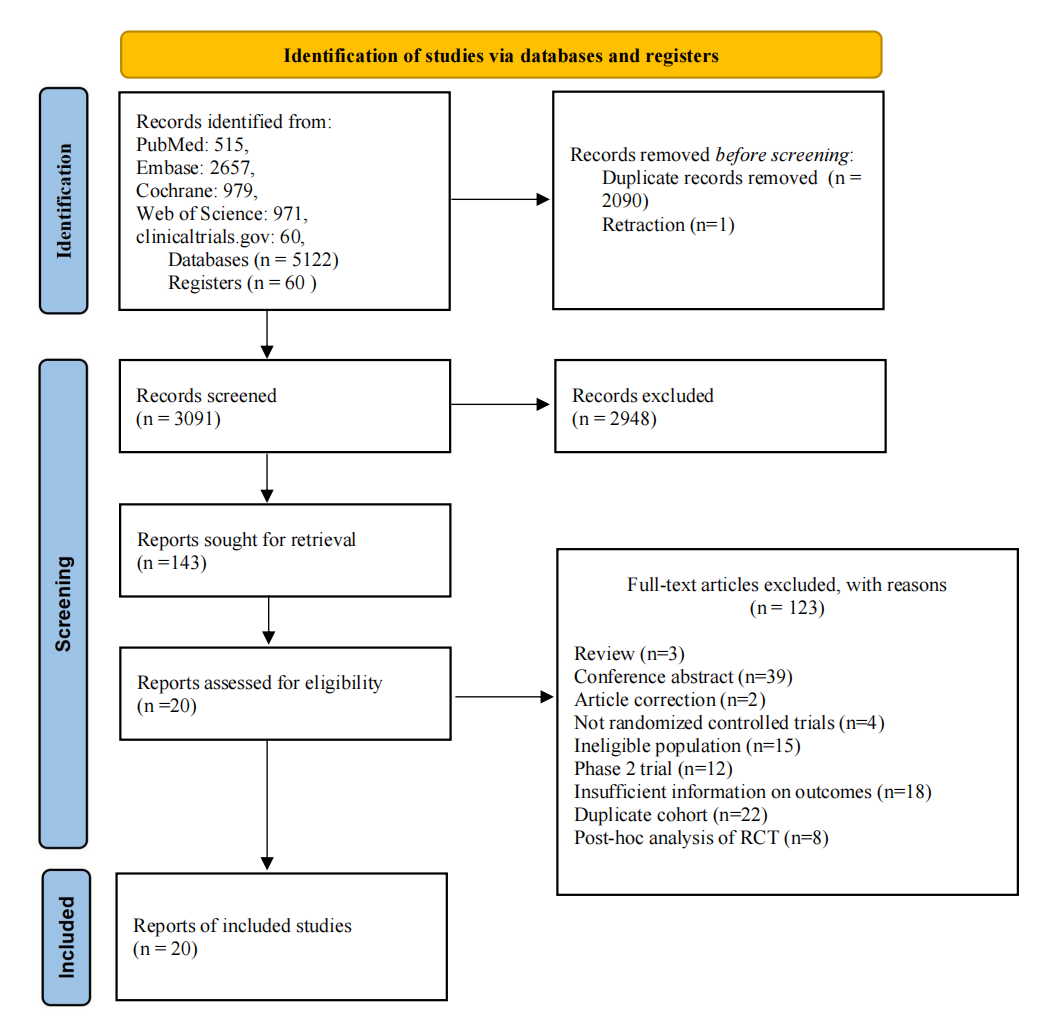


**Supplementary figure 1** Flow diagram of assessment of studies identified in the systematic review.


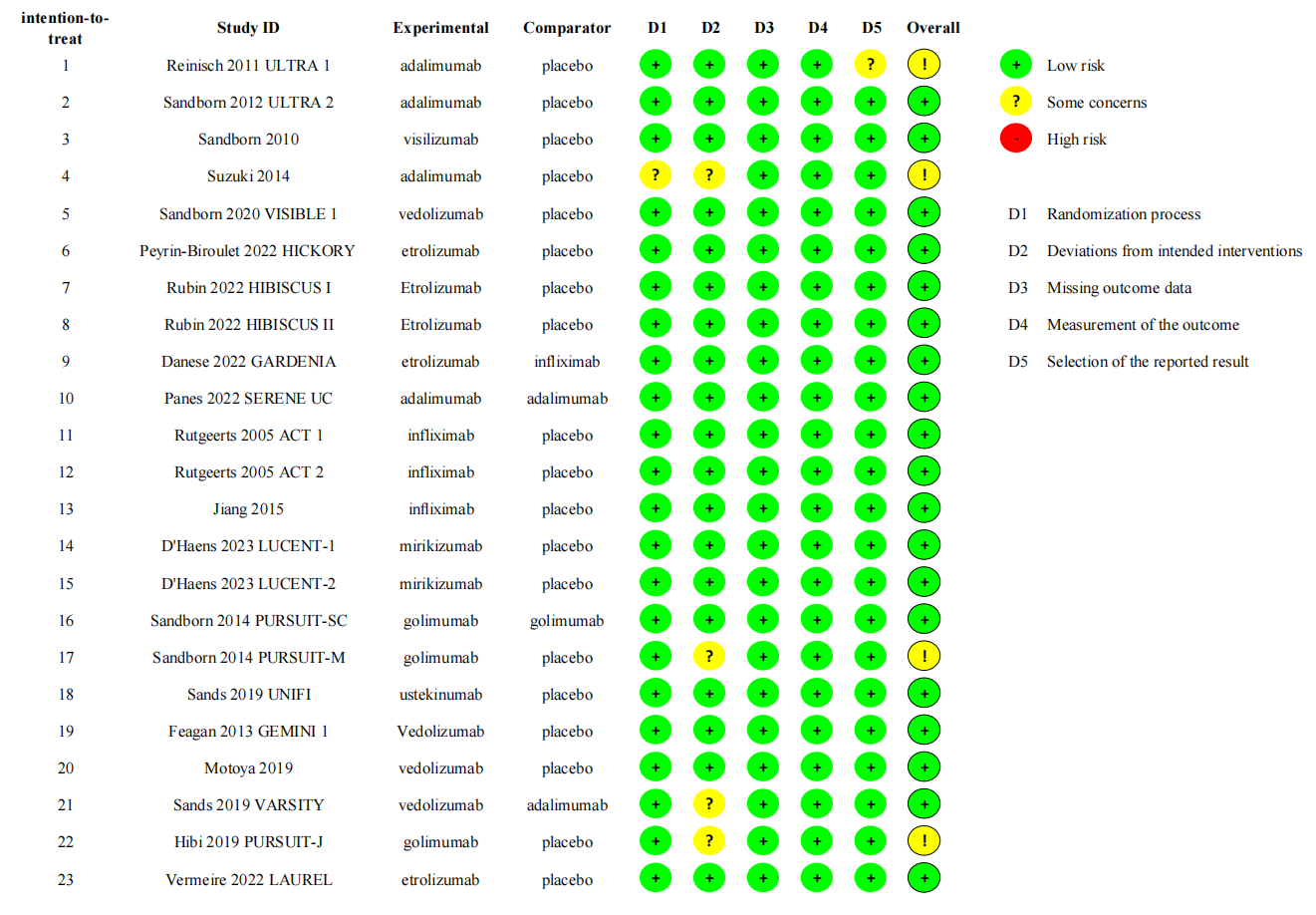


**Supplementary figure 2** Risk of bias of RCTs of biological therapy in moderate to severe UC.

| A  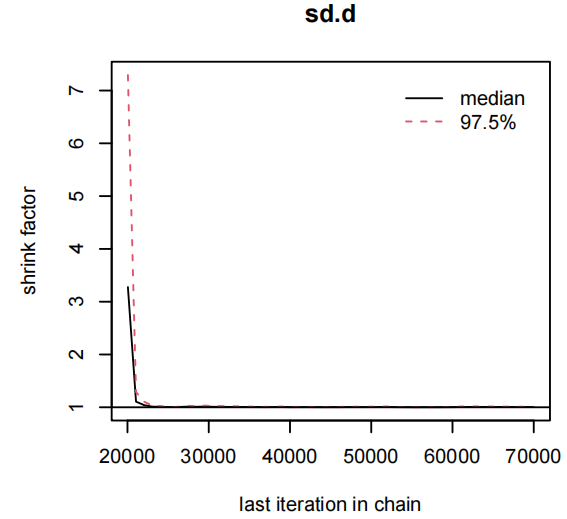 | B  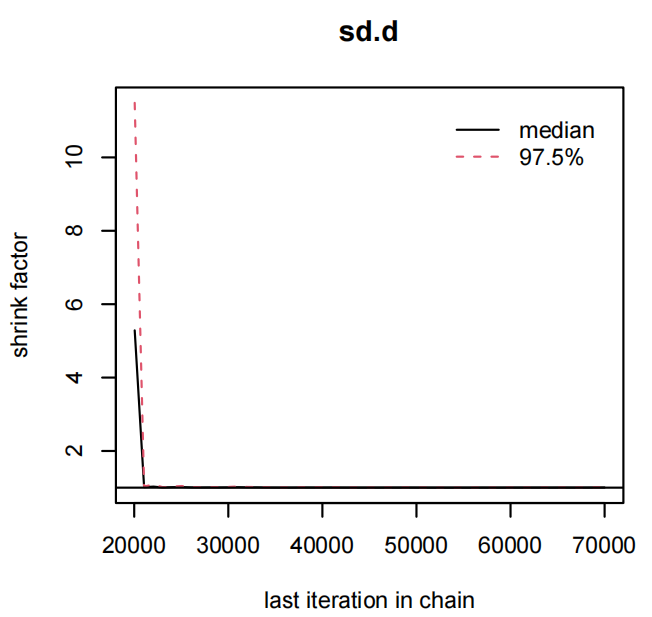 |
| --- | --- |
| 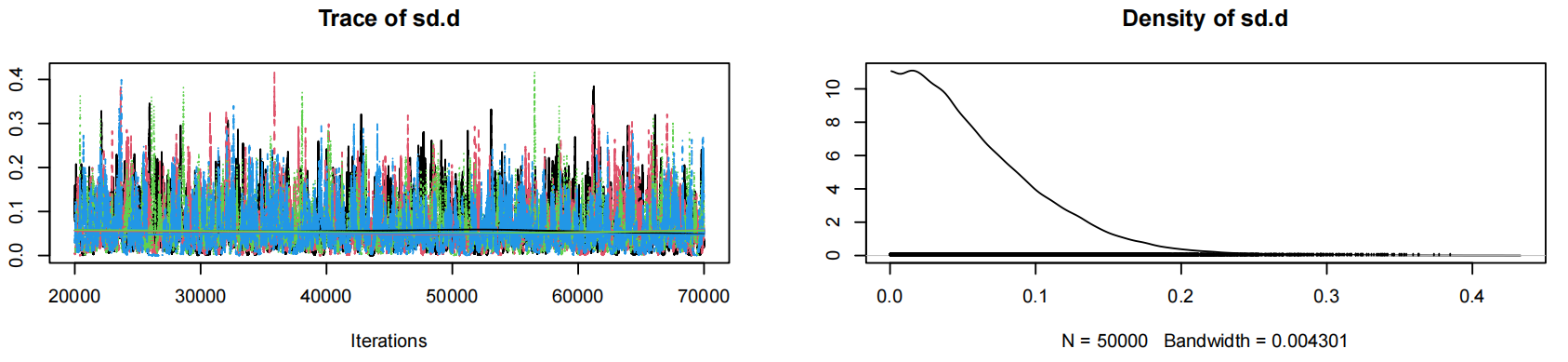 | 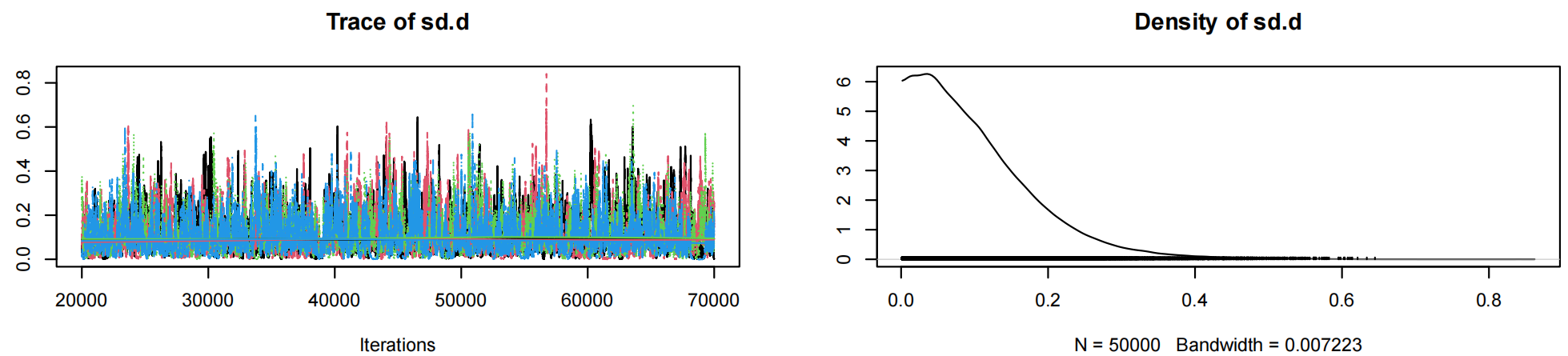 |
| **Supplementary figure 3** Potential scale reduction factor (PSRF) for endoscopic improvement in (A) and (C): induction therapy, (B) and (D): maintenance therapy. | |


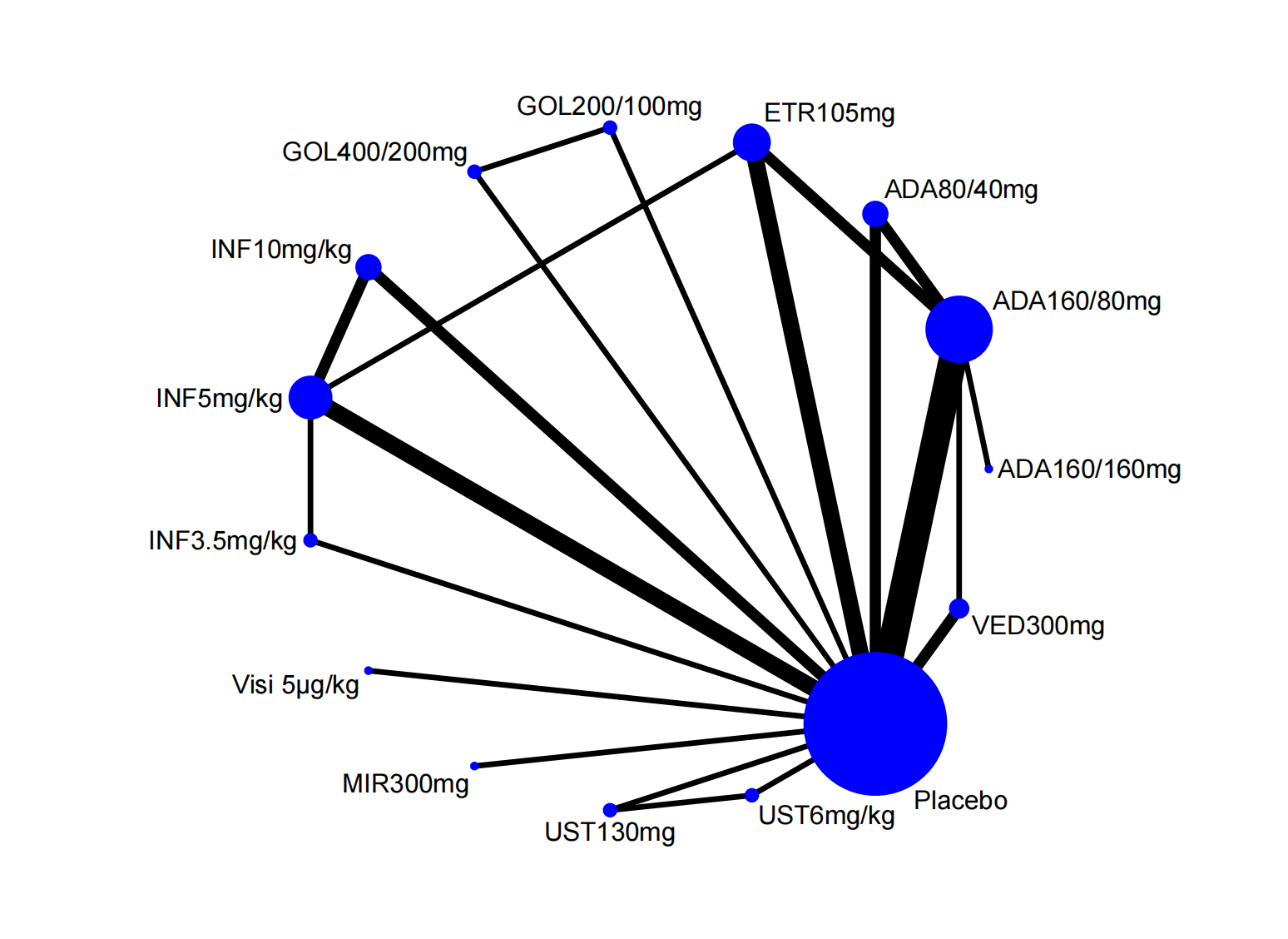


**Supplementary figure 4** Network graphs for outcome of clinical remission in induction therapy.

ADA, adalimumab; ETR, etrolizumab; GOL, golimumab; IFX, infliximab; UST, ustekinumab; VED, vedolizumab; MIR, mirikizumab；Visi, visilizumab.

**Supplementary figure 5** SUCRA values for induction therapy of the efficacy outcomes: all patients.

ADA, adalimumab; ETR, etrolizumab; GOL, golimumab; IFX, infliximab; UST, ustekinumab; VED, vedolizumab; MIR, mirikizumab；Visi, visilizumab.

**Supplementary figure 6** SUCRA values for maintenance therapy of the efficacy outcomes: all patients.

Note: ADA, adalimumab; ETR, etrolizumab; GOL, golimumab; IFX, infliximab; UST, ustekinumab; VED, vedolizumab; MIR, mirikizumab; EOW, every other week; QW, every week; Q12W, every 12weeks; Q8W, every 8 weeks; Q4W, every 4 weeks.

**Supplementary figure 7** SUCRA values for induction therapy of the efficacy outcomes: biologic-naive patients.

Note: ADA, adalimumab; ETR, etrolizumab; GOL, golimumab; IFX, infliximab; UST, ustekinumab; VED, vedolizumab; MIR, mirikizumab.

**Supplementary figure 8** SUCRA values for induction therapy of the efficacy outcomes: biologic-exposed patients.

Note: ADA, adalimumab; ETR, etrolizumab; UST, ustekinumab; VED, vedolizumab; MIR, mirikizumab.


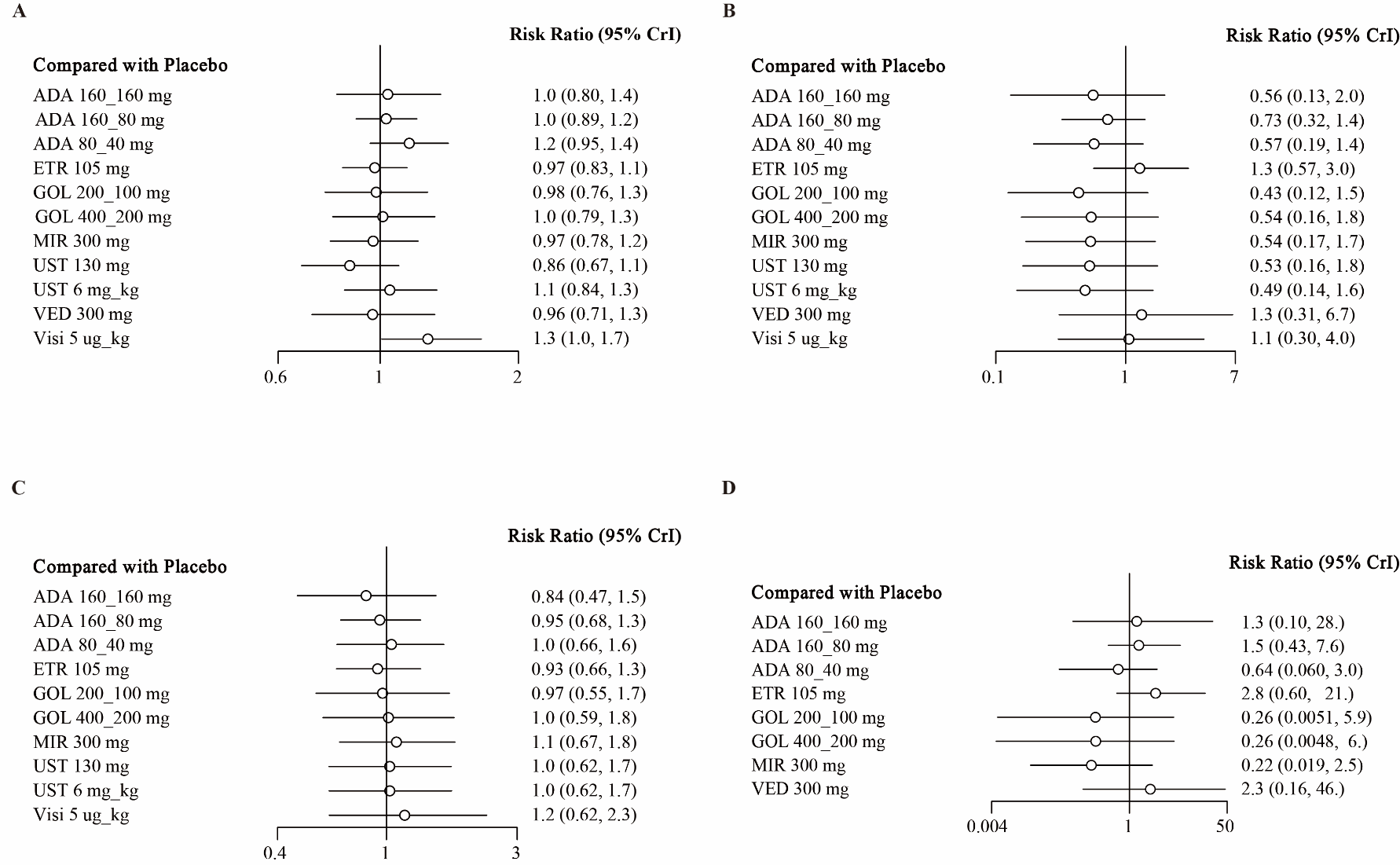


**Supplementary figure 9** Forest plot for evaluating safety outcomes in induction therapy:

(A) total adverse events; (B) serious adverse events; (C) infections; (D) adverse events leading to withdrawal.

Note: ADA, adalimumab; ETR, etrolizumab; GOL, golimumab; IFX, infliximab; UST, ustekinumab; VED, vedolizumab; MIR, mirikizumab; Visi, visilizumab; EOW, every other week; QW, every week; Q12W, every 12 weeks; Q8W, every 8 weeks.

**Supplementary figure 10** SUCRA values for induction therapy of the safety outcomes: all patients.

Note: ADA, adalimumab; ETR, etrolizumab; GOL, golimumab; IFX, infliximab; UST, ustekinumab; VED, vedolizumab; MIR, mirikizumab; Visi, visilizumab. Withdrawal, adverse events leading to withdrawal; AE, adverse events; SAE, serious adverse events.

**Supplementary figure 11** SUCRA values for maintenance therapy of the safety outcomes: all patients.

Note: ADA, adalimumab; ETR, etrolizumab; GOL, golimumab; IFX, infliximab; UST, ustekinumab; VED, vedolizumab; MIR, mirikizumab; EOW, every other week; QW, every week; Q12W, every 12weeks; Q8W, every 8 weeks. Withdrawal, adverse events leading to withdrawal; AE, adverse events; SAE, serious adverse events.


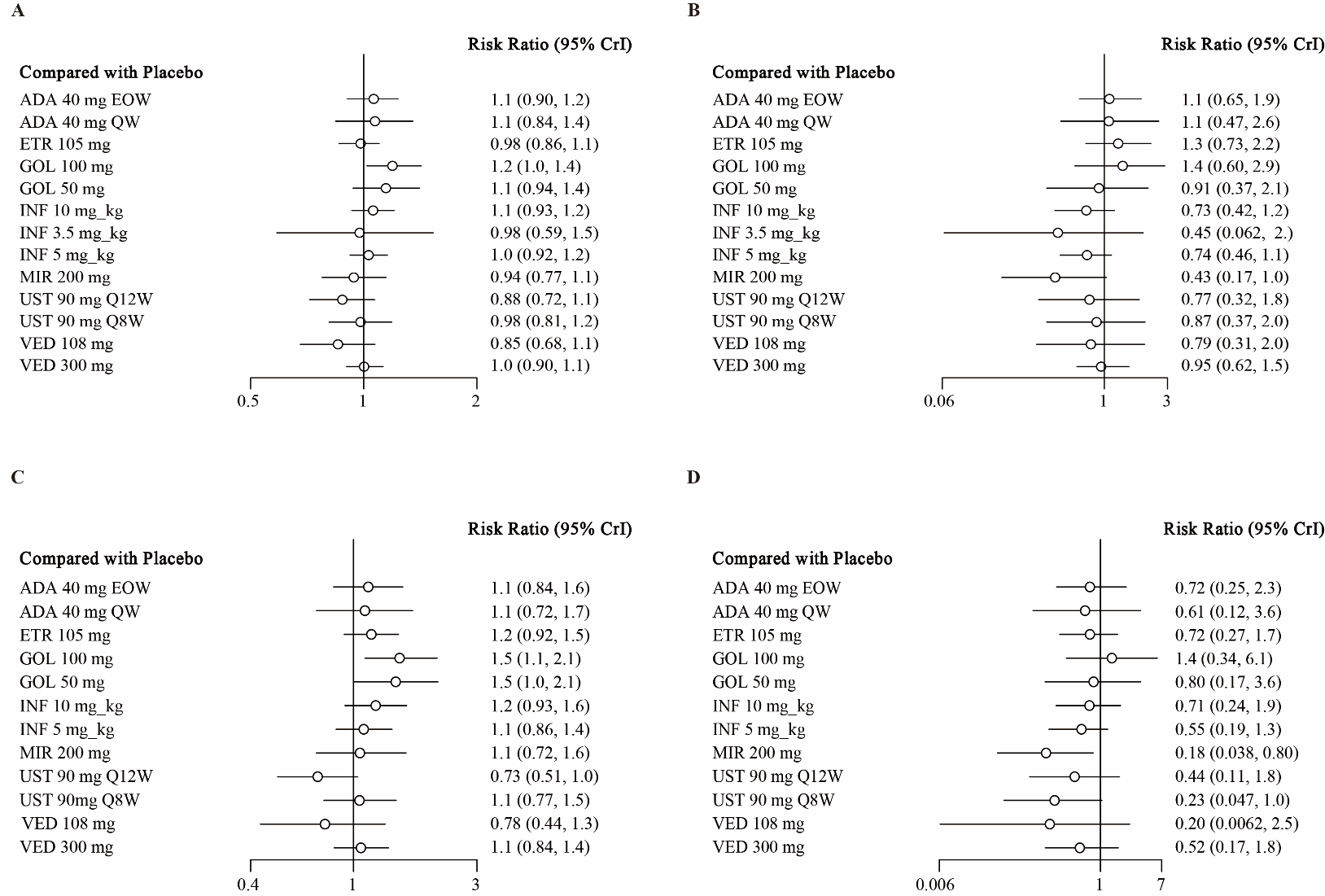


**Supplementary figure 12** Forest plot for evaluating safety outcomes in maintenance therapy:

(A) total adverse events; (B) serious adverse events; (C) infections; (D) adverse events leading to withdrawal.

Note: ADA, adalimumab; ETR, etrolizumab; GOL, golimumab; IFX, infliximab; UST, ustekinumab; VED, vedolizumab; MIR, mirikizumab; Visi, visilizumab; EOW, every other week; QW, every week; Q12W, every 12 weeks; Q8W, every 8 weeks.

**Supplementary table 1**  Reported Endpoints in RCTs of Biological Therapies in Moderate to Severe UC.

| **Study** | **Clinical Remission** | **Endoscopic Improvement** | **Clinical Response** |
| --- | --- | --- | --- |
| **Reinisch 2011 ULTRA 1** | Mayo score ≤2, with no subscore >1 | Mayo endoscopic subscore of ≤1 | Decrease in Mayo score by ≥3 and 30% and a decrease in rectal bleeding subscore of ≥1 or an absolute subscore for rectal bleeding of 0 or 1 |
| **Sandborn 2012 ULTRA 2** | Mayo score ≤2, with no subscore >1 | Mayo endoscopic subscore of ≤1 | Decrease in Mayo score by ≥3 and 30% and a decrease in rectal bleeding subscore of ≥1 or an absolute subscore for rectal bleeding of 0 or 1 |
| **Sandborn 2010** | Mayo score ≤2, with no subscore >1 | Mayo endoscopic subscore of ≤1 | Decrease in Mayo score by ≥3 and 30% and a decrease in rectal bleeding subscore of ≥1 or an absolute subscore for rectal bleeding of 0 or 1 |
| **Suzuki 2014** | Mayo score ≤2, with no subscore >1 | Mayo endoscopic subscore of ≤1 | Decrease in Mayo score by ≥3 and 30% and a decrease in rectal bleeding subscore of ≥1 or an absolute subscore for rectal bleeding of 0 or 1 |
| **Sandborn 2020 VISIBLE 1** | Mayo score ≤2, with no subscore >1 | Mayo endoscopic subscore of ≤1 | Decrease in Mayo score by ≥3 and 30% and a decrease in rectal bleeding subscore of ≥1 or an absolute subscore for rectal bleeding of 0 or 1 |
| **Peyrin-Birouet 2021 HICKORY** | Mayo score ≤2, with no subscore >1 and a rectal bleeding subscore of 0 | Mayo endoscopic subscore of ≤1 | Decrease in Mayo score by ≥3 and 30% and a decrease in rectal bleeding subscore of ≥1 or an absolute subscore for rectal bleeding of 0 or 1 |
| **Rubin 2021 HIBISCUS I** | Mayo score ≤2, with no subscore >1 and a rectal bleeding subscore of 0 | Mayo endoscopic subscore of ≤1 | Decrease in Mayo score by ≥3 and 30% and a decrease in rectal bleeding score of ≥1 or an absolute subscore for rectal bleeding of 0 or 1 |
| **Rubin 2021 HIBISCUS II** | Mayo score ≤2, with no subscore >1 and a rectal bleeding subscore of 0 | Mayo endoscopic subscore of ≤1 | Decrease in Mayo score by ≥3 and 30% and a decrease in rectal bleeding score of ≥1 or an absolute subscore for rectal bleeding of 0 or 1 |
| **Danese 2021 GARDENIA** | Mayo score ≤2, with no subscore >1 | Mayo endoscopic subscore of ≤1 | Decrease in Mayo score by ≥3 and 30% and a decrease in rectal bleeding subscore of ≥1 or an absolute subscore for rectal bleeding of 0 or 1 |
| **Panes 2019 SERENE-UC** | Mayo score ≤2, with no subscore >1 | Mayo endoscopic subscore of ≤1 | Decrease in Mayo score by ≥3 and 30% and a decrease in rectal bleeding subscore of ≥1 or an absolute subscore for rectal bleeding of 0 or 1 |
| **Rutgeerts 2005 ACT 1** | Mayo score ≤2, with no subscore >1 | Mayo endoscopic subscore of ≤1 | Decrease in Mayo score by ≥3 and 30% and a decrease in rectal bleeding subscore of ≥1 or an absolute subscore for rectal bleeding of 0 or 1 |
| **Rutgeerts 2005 ACT 2** | Mayo score ≤2, with no subscore >1 | Mayo endoscopic subscore of ≤1 | Decrease in Mayo score by ≥3 and 30% and a decrease in rectal bleeding subscore of ≥1 or an absolute subscore for rectal bleeding of 0 or 1 |
| **Jiang 2015** | Mayo score ≤2, with no subscore >1 | Mayo endoscopic subscore of ≤1 | Decrease in Mayo score by ≥3 and 30% and a decrease in rectal bleeding subscore of ≥1 or an absolute subscore for rectal bleeding of 0 or 1 |
| **G. D'Haens 2023 LUCENT-1** | Modified Mayo stool-frequency subscore of 0 or a stool-frequency subscore of 1 with a decrease of at least 1 point from baseline, a rectal-bleeding subscore of 0, and an endoscopic subscore of 0 or 1 (excluding friability). | Mayo endoscopic subscore of ≤1 | Decreases of ≥2 points and ≥30% from baseline in the modified Mayo score, plus a rectal-bleeding subscore of 0 or 1 or a decrease of ≥1 point from baseline |
| **G. D'Haens 2023 LUCENT-2** | Modified Mayo stool-frequency subscore of 0 or a stool-frequency subscore of 1 with a decrease of at least 1 point from baseline, a rectal-bleeding subscore of 0, and an endoscopic subscore of 0 or 1 (excluding friability). | Mayo endoscopic subscore of ≤1 | Decreases of ≥2 points and ≥30% from baseline in the modified Mayo score, plus a rectal-bleeding subscore of 0 or 1 or a decrease of ≥1 point from baseline |
| **Sandborn 2014 PURSUIT-SC** | Mayo score ≤2, with no subscore >1 | Mayo endoscopic subscore of ≤1 | Decrease in Mayo score by ≥3 and 30% and a decrease in rectal bleeding subscore of ≥1 or an absolute subscore for rectal bleeding of 0 or 1 |
| **Sandborn 2014 PURSUIT M** | Mayo score ≤2, with no subscore >1 | Mayo endoscopic subscore of ≤1 | Decrease in Mayo score by ≥3 and 30% and a decrease in rectal bleeding subscore of ≥1 or an absolute subscore for rectal bleeding of 0 or 1 |
| **Sands 2019 UNIFI** | Mayo score ≤2, with no subscore >1 | Mayo endoscopic subscore of ≤1 | Decrease in Mayo score by ≥3 and 30% and a decrease in rectal bleeding subscore of ≥1 or an absolute subscore for rectal bleeding of 0 or 1 |
| **Feagan 2013 GEMINI 1** | Mayo score ≤2, with no subscore >1 | Mayo endoscopic subscore of ≤1 | Decrease in Mayo score by ≥3 and 30% and a decrease in rectal bleeding subscore of ≥1 or an absolute subscore for rectal bleeding of 0 or 1 |
| **Motoya 2019** | Mayo score ≤2, with no subscore >1 | Mayo endoscopic subscore of ≤1 | Decrease in Mayo score by ≥3 and 30% and a decrease in rectal bleeding subscore of ≥1 or an absolute subscore for rectal bleeding of 0 or 1 |
| **Sands 2019 VARSITY** | Mayo score ≤2, with no subscore >1 | Mayo endoscopic subscore of ≤1 | Decrease in Mayo score by ≥2 and 25% and a decrease in rectal bleeding subscore of ≥1 or an absolute subscore for rectal bleeding of 0 or 1 |
| **Hibi 2017 PURSUIT J** | Mayo score ≤2, with no subscore >1 | Mayo endoscopic subscore of ≤1 | Decrease in Mayo score by ≥2 and 25% and a decrease in rectal bleeding subscore of ≥1 or an absolute subscore for rectal bleeding of 0 or 1 |
| **Vermeire 2021 LAUREL** | Mayo score ≤2, with no subscore >1 | Mayo endoscopic subscore of ≤1 | Decrease in Mayo score by ≥2 and 25% and a decrease in rectal bleeding subscore of ≥1 or an absolute subscore for rectal bleeding of 0 or 1 |

**Supplementary table 2**  Characteristics of included studies of biological therapies in moderate to severe UC.

| **Study** | **Number of Patients** | **Medication (Experiment)**  **(Number of patients)** | **Medication (Control)**  **(Number of patients)** | **Disease duration**  **(Experiment) (Year)** | **Disease duration**  **(Control) (Year)** | **Duration of**  **Follow-up** **(Week)** | **Racial**  **Composition^#^** |
| --- | --- | --- | --- | --- | --- | --- | --- |
| **Reinisch 2011 ULTRA 1** | 390 | Adalimumab 80mg at week 0, 40 mg at week 2, 4, 6. (130)  Adalimumab 160 at week 0, 80mg at week 2, 40 mg at week 4, 6. (130) | Placebo (130) | 6.91 (0.3-39.7)*  5.35 (0.3-34.1)* | 6.06 (0.2-34.4)* | 8 | Not reported |
| **Sandborn 2012**  **ULTRA 2** | 494 | Adalimumab 160 at week 0, 80mg at week 2, then 40mg every other week. (248) | Placebo (246) | 8.1±7.09 | 8.5±7.37 | 52 | Not reported |
| **Sandborn 2010** | 127 | Visilizumab 5μg/kg on day 1 and 2 (84) | Placebo (43) | 6.8±7.1 | 6.4±6.8 | 45 days | Not reported |
| **Suzuki 2014** | 273 | Adalimumab 80mg at week 0, 40 mg at week 2, then 40mg every other week. (87)  Adalimumab 160 at week 0, 80mg at week 2, then 40mg every other week. (90) | Placebo (96) | 8.3±7.7  7.8±7.1 | 7.8±6.6 | 8/52 | Not reported |
| **Sandborn 2020 VISIBLE 1** | 216 | Vedolizumab 108mg, SC, every other week. (106)  Vedolizumab 300 mg, IV, every 8 weeks. (54) | Placebo (56) | 8.0±6.2  8.2±5.9 | 7.4±7.1 | 52 | A: 32  B: 2  W: 181  O: 1 |
| **Peyrin-Birouet 2022 HICKORY** | 479 | Etrolizumab 105mg every 4 weeks. (384) | Placebo (95) | 7.10 (0.6–44.0)* | 7.36 (0.8–40.9)* | 14/66 | A: 31  B: 7  W: 377  O: 64 |
| **Rubin 2021 HIBISCUS I** | 358 | Etrolizumab 105mg every 4 weeks. (144)  Adalimumab 160 at week 0, 80mg at week 2, then 40mg every other week. (142) | Placebo (72) | 3.4 (0.4–41.9)*  4.0(0.3–36.4)* | 4.7 (0.3–40.8)* | 14 | A: 2  B: 3  W: 336  O: 17 |
| **Rubin 2021 HIBISCUS II** | 358 | Etrolizumab 105mg every 4 weeks. (143)  Adalimumab 160 at week 0, 80mg at week 2, then 40mg every other week. (143) | Placebo (72) | 3.6 (0.3–58.8)*  4.1 (0.3–37.9)* | 4.0 (0.3–25.4)* | 14 | A: 12  B: 6  W: 329  O: 11 |
| **Danese 2021 GARDENIA** | 397 | Etrolizumab 105mg every 4 weeks. (199) | Infliximab 5mg/kg at week 0, 2, and 6, then every 8 weeks (198). | 3∙3 (0∙3–49∙0)* | 4∙1 (0∙3–33∙7)* | 10/54 | A: 69  B: 1  W: 308  O: 19 |
| **Panes 2019 SERENE-UC** | 952 | Adalimumab 160mg at week 0, 1, 2, and 3, then 40mg every week. (573) | Adalimumab 160 at week 0, 80mg at week 2, then 40mg every other week. (379) | 7.2±7.2 | 7.0±7.0 | 8/52 | A: 114  B: 24  W: 810  O: 3  N:1 |
| **Rutgeerts 2005 ACT 1** | 364 | Infliximab 5mg/kg at week 0, 2, and 6, then every 8 weeks. (121)  Infliximab 10mg/kg at week 0, 2, and 6, then every 8 weeks. (122) | Placebo (121) | 5.9±5.4  8.4±8.1 | 6.2±5.9 | 8/54 | A: Not reported  B: Not reported  W: 340  O: Not reported |
| **Rutgeerts 2005 ACT 2** | 364 | Infliximab 5mg/kg at week 0, 2, and 6, then every 8 weeks. (121)  Infliximab 10mg/kg at week 0, 2, and 6, then every 8 weeks. (120) | Placebo (123) | 6.7±5.3  6.5±5.8 | 6.5±6.7 | 8/54 | A: Not reported  B: Not reported  W: 344  O: Not reported |
| **Jiang 2015** | 123 | Infliximab 3.5mg/kg at week 0, 2, and 6, then every 8 weeks. (41)  Infliximab 5mg/kg at week 0, 2, and 6, then every 8 weeks. (41) | Placebo (41) | 4.3±2.5  4.4±2.8 | 4.4±2.6 | 8/30 | Not reported |
| **G. D'Haens 2023 LUCENT-1** | 1281 | Mirikizumab 300mg at week 0, 4, and 8. (959) | Placebo (322) | 7.2±6.7 | 6.9±7.0 | 12 | A: 292  B: 12  W: 951  O: 16  N: 10 |
| **G. D'Haens 2023 LUCENT-2** | 581 | Mirikizumab 200mg every 4 weeks. (389) | Placebo (192) | 7.2±6.7 | 6.9±7.0 | 40 | A: 172  B: 6  W: 526  O: 8  N: 4 |
| **Sandborn 2014 PURSUIT-SC** | 993 | Golimumab 400 at week 0 and 200mg at week 2 (331)  Golimumab 200 at week 0 and 100mg at week 2. (331) | Placebo (331) | 6.4±6.27  6.4±6.17 | 6.0±6.65 | 6 | A: 122  B: 26  W: 809  O: 36 |
| **Sandborn 2014 PURSUIT M** | 464 | Golimumab 50mg every 4 weeks. (154)  Golimumab 100mg every 4 weeks. (154) | Placebo (156) | 6.8±6.93  7.2±7.04 | 6.9±6.96 | 52 | A: 38  B: 8  W: 405  O: 13 |
| **Sands 2019 UNIFI** | 961 | Ustekinumab 130mg at week 0,then 90mg every 12 week. (320)  Ustekinumab 6mg/kg at week 0, then 90mg every 8 week. (322) | Placebo (319) | 8.1±7.2  8.2±7.8 | 8.0±7.2 | 8/44 | A: 143  B: 9  W: 730  O: 30  N: 49 |
| **Feagan 2013 GEMINI 1** | 374 | Vedolizumab 300mg at week 0 and 2, then 300mg every 8 week. (225) | Placebo (149) | 6.1±5.1 | 7.1±7.2 | 8/52 | A: 68  B: 7  W: 298  O: 1 |
| **Motoya 2019** | 246 | Vedolizumab 300mg at week 0, 2, and 6, then every 8 weeks. (164) | Placebo (82) | 7.2±6.2 | 8.6±8.0 | 10/60 | Not reported |
| **Sands 2019 VARSITY** | 771 | Adalimumab 160 at week 0, 80mg at week 2, then 40mg every other week. (386) | Vedolizumab 300mg at week 0, 2, and 6, then every 8 weeks. (385) | 6.4±6.0 | 7.3±7.2 | 14/52 | A: 62  B: 5  W: 686  O: 18 |
| **Hibi 2017 PURSUIT J** | 63 | Golimumab 100mg every 4 weeks. (32) | Placebo (31) | 5.35 (0.5-24.7)* | 5.74 (0.3-21.6)* | 52 | Not reported |
| **Vermeire 2021 LAUREL** | 210 | Etrolizumab 105mg every 4 weeks. (108) | Placebo (102) | 5·4 (0·6–44·0)* | 5·9 (0·3–40·4)* | 52 | A: 34  B: 8  W: 157  O: 4  N: 11 |

Values are given in mean and standard deviation unless otherwise stated.

* Values given in median and range

# A: Asian; B: Black or African America; W: White; O: Other; N: Unknown or not reported.

**Supplementary table 3** Indirect comparison for the subset of biologic-naive patients and biologic-exposed patients for achieving clinical remission。

| ADA  160/80 mg |  | 3.17  (0.12, 80.98) |  |  |  |  |  | 1.88  (0.08, 45.98) | 1.01  (0.13, 7.8) | 12  (0.41, 19.49) | 13.84  (0.47, 470.65) | 1.5  (0.19, 10.87) | Biologic-exposed patients |
| --- | --- | --- | --- | --- | --- | --- | --- | --- | --- | --- | --- | --- | --- |
| 1.47  (0.88, 2.53) | ADA  80/40 mg |  |  |  |  |  |  |  |  |  |  |  |  |
| 1.12  (0.76, 1.62) | 0.76  (0.4, 1.39) | ETR  105 mg |  |  |  |  |  | 0.6  (0.02, 19.3) | 0.32  (0.03, 3.75) | 3.76  (0.1, 167.04) | 4.33  (0.11, 195.8) | 0.47  (0.02, 11.54) |  |
| 0.7  (0.32, 1.45) | 0.47  (0.19, 1.12) | 0.62  (0.28, 1.36) | GOL  200/100 mg |  |  |  |  |  |  |  |  |  |  |
| 0.69  (0.32, 1.44) | 0.47  (0.19, 1.12) | 0.62  (0.28, 1.36) | 1  (0.57, 1.72) | GOL  400/200 mg |  |  |  |  |  |  |  |  |  |
| 0.77  (0.45, 1.26) | 0.52  (0.25, 1.01) | 0.69  (0.4, 1.12) | 1.11  (0.49, 2.44) | 1.11  (0.48, 2.47) | INF  10 mg/kg |  |  |  |  |  |  |  |  |
| 0.73  (0.37, 1.4) | 0.5  (0.22, 1.09) | 0.65  (0.34, 1.25) | 1.05  (0.43, 2.61) | 1.05  (0.43, 2.63) | 0.94  (0.5, 1.85) | INF  3.5 mg/kg |  |  |  |  |  |  |  |
| 0.65  (0.41, 0.99) | 0.44  (0.23, 0.82) | 0.58  (0.38, 0.86) | 0.94  (0.43, 2.02) | 0.94  (0.43, 2.02) | 0.85  (0.59, 1.24) | 0.9  (0.51, 1.52) | INF  5 mg/kg |  |  |  |  |  |  |
| 1.01  (0.52, 1.88) | 0.69  (0.31, 1.47) | 0.9  (0.46, 1.76) | 1.44  (0.6, 3.48) | 1.46  (0.6, 3.52) | 1.3  (0.65, 2.71) | 1.38  (0.61, 3.1) | 1.54  (0.81, 3.02) | MIR  300 mg | 0.54  (0.05, 6.16) | 6.27  (0.17, 278.28) | 7.25  (0.2, 324.24) | 0.79  (0.03, 19.33) |  |
| 1.98  (1.44, 2.74) | 1.34  (0.76, 2.32) | 1.77  (1.21, 2.63) | 2.84  (1.46, 5.72) | 2.86  (1.48, 5.7) | 2.58  (1.69, 4.11) | 2.72  (1.5, 4.98) | 3.03  (2.16, 4.44) | 1.97  (1.15, 3.47) | Placebo | 11.63  (0.82, 213.03) | 13.45  (0.97, 244.87) | 1.47  (0.17, 11.68) |  |
| 0.93  (0.42, 2) | 0.64  (0.25, 1.52) | 0.83  (0.37, 1.85) | 1.34  (0.5, 3.61) | 1.34  (0.51, 3.62) | 1.21  (0.53, 2.83) | 1.28  (0.5, 3.23) | 1.43  (0.65, 3.2) | 0.93  (0.38, 2.26) | 0.47  (0.23, 0.94) | UST  130 mg | 1.16  (0.1, 13.35) | 0.13  (0, 3.64) |  |
| 1.05  (0.47, 2.31) | 0.72  (0.28, 1.75) | 0.93  (0.41, 2.15) | 1.51  (0.57, 4.15) | 1.51  (0.57, 4.13) | 1.36  (0.59, 3.25) | 1.44  (0.56, 3.67) | 1.6  (0.72, 3.66) | 1.04  (0.42, 2.6) | 0.53  (0.25, 1.08) | 1.13  (0.61, 2.09) | UST  6 mg/kg | 0.11  (0, 3.03) |  |
| 0.88  (0.57, 1.37) | 0.6  (0.31, 1.16) | 0.78  (0.46, 1.4) | 1.27  (0.56, 3) | 1.27  (0.56, 3.02) | 1.14  (0.62, 2.27) | 1.2  (0.57, 2.64) | 1.34  (0.77, 2.49) | 0.87  (0.42, 1.88) | 0.44  (0.27, 0.73) | 0.95  (0.4, 2.29) | 0.84  (0.35, 2.03) | VED  300 mg |  |
| Biologic-naive patients | | | | | | | | | | | | |  |

Relative risk with 95% confidence intervals in parentheses. Comparisons between columns and rows should be read from left to right. The blue boxes represent statistically significant

comparisons and the white boxes represent non-statistically significant comparisons.

Note: ADA, adalimumab; ETR, etrolizumab; GOL, golimumab; IFX, infliximab; UST, ustekinumab; VED, vedolizumab; MIR, mirikizumab.

**Supplementary table 4**  Indirect comparison for the subset of biologic-naive patients and biologic-exposed patients for achieving clinical response.

| ADA  160/80 mg |  | 1.38  (0.43, 4.55) |  |  |  |  |  | 1.73  (0.55, 5.67) | 0.93  (0.45, 1.98) | 1.56  (0.49, 5.15) | 1.98  (0.63, 6.48) | 1.47  (0.69, 3.03) | Biologic-exposed patients |
| --- | --- | --- | --- | --- | --- | --- | --- | --- | --- | --- | --- | --- | --- |
| 1.12  (0.89, 1.4) | ADA  80/40 mg |  |  |  |  |  |  |  |  |  |  |  |  |
| 0.95  (0.79, 1.14) | 0.85  (0.65, 1.12) | ETR  105 mg |  |  |  |  |  | 1.25  (0.34, 4.6) | 0.68  (0.27, 1.69) | 1.13  (0.31, 4.18) | 1.43  (0.39, 5.23) | 1.07  (0.34, 3.15) |  |
| 0.79  (0.56, 1.1) | 0.71  (0.48, 1.03) | 0.83  (0.58, 1.18) | GOL  200/100 mg |  |  |  |  |  | 0.54  (0.22, 1.33) | 0.9  (0.25, 3.3) | 1.14  (0.32, 4.11) | 0.85  (0.27, 2.5) |  |
| 0.73  (0.52, 1.02) | 0.66  (0.45, 0.95) | 0.77  (0.54, 1.09) | 0.93  (0.71, 1.22) | GOL  400/200 mg |  |  |  |  |  | 1.67  (0.67, 4.2) | 2.12  (0.85, 5.29) | 1.58  (0.81, 2.91) |  |
| 0.71  (0.55, 0.9) | 0.64  (0.46, 0.86) | 0.75  (0.57, 0.95) | 0.91  (0.62, 1.3) | 0.97  (0.67, 1.4) | INF  10 mg/kg |  |  |  |  |  | 1.27  (0.51, 3.1) | 0.95  (0.3, 2.78) |  |
| 0.74  (0.51, 1.06) | 0.67  (0.44, 0.99) | 0.78  (0.54, 1.12) | 0.94  (0.59, 1.48) | 1.01  (0.64, 1.59) | 1.04  (0.73, 1.5) | INF  3.5 mg/kg |  |  |  |  |  | 0.75  (0.24, 2.18) |  |
| 0.71  (0.56, 0.87) | 0.63  (0.47, 0.84) | 0.75  (0.6, 0.91) | 0.9  (0.63, 1.27) | 0.97  (0.68, 1.37) | 0.99  (0.82, 1.2) | 0.95  (0.69, 1.31) | INF  5 mg/kg |  |  |  |  |  |  |
| 0.95  (0.7, 1.28) | 0.85  (0.6, 1.2) | 1  (0.72, 1.37) | 1.21  (0.81, 1.8) | 1.3  (0.87, 1.94) | 1.33  (0.96, 1.89) | 1.28  (0.83, 1.99) | 1.34  (0.98, 1.86) | MIR  300 mg |  |  |  |  |  |
| 1.33  (1.15, 1.54) | 1.19  (0.94, 1.5) | 1.41  (1.16, 1.68) | 1.69  (1.25, 2.3) | 1.82  (1.35, 2.46) | 1.87  (1.52, 2.35) | 1.8  (1.27, 2.54) | 1.88  (1.58, 2.27) | 1.4  (1.08, 1.83) | Placebo |  |  |  |  |
| 0.82  (0.57, 1.17) | 0.73  (0.49, 1.09) | 0.86  (0.59, 1.25) | 1.04  (0.66, 1.62) | 1.12  (0.72, 1.74) | 1.15  (0.78, 1.71) | 1.1  (0.69, 1.78) | 1.15  (0.8, 1.69) | 0.86  (0.56, 1.31) | 0.61  (0.44, 0.85) | UST  130 mg |  |  |  |
| 0.71  (0.5, 1) | 0.64  (0.43, 0.93) | 0.75  (0.51, 1.07) | 0.9  (0.58, 1.4) | 0.97  (0.63, 1.5) | 1  (0.68, 1.47) | 0.95  (0.6, 1.54) | 1  (0.7, 1.45) | 0.75  (0.49, 1.13) | 0.53  (0.39, 0.73) | 0.87  (0.66, 1.15) | UST  6 mg/kg |  |  |
| 0.73  (0.59, 0.93) | 0.66  (0.49, 0.91) | 0.78  (0.59, 1.04) | 0.93  (0.64, 1.42) | 1  (0.69, 1.52) | 1.03  (0.76, 1.47) | 0.99  (0.66, 1.55) | 1.04  (0.78, 1.44) | 0.77  (0.55, 1.14) | 0.55  (0.43, 0.73) | 0.9  (0.6, 1.39) | 1.03  (0.7, 1.59) | VED  300 mg |  |
| Biologic-naive patients | | | | | | | | | | | | |  |

Relative risk with 95% confidence intervals in parentheses. Comparisons between columns and rows should be read from left to right. The blue boxes represent statistically significant

comparisons and the white boxes represent non-statistically significant comparisons.

Note: ADA, adalimumab; ETR, etrolizumab; GOL, golimumab; IFX, infliximab; UST, ustekinumab; VED, vedolizumab; MIR, mirikizumab.

**Supplementary table 5** Indirect comparison for the subset of biologic-naive patients and biologic-exposed patients for achieving endoscopic improvement.

| ADA  160/80 mg |  | 1.25  (0.17, 9.25) |  |  |  |  |  | 2.24  (0.3, 17.06) | 0.94  (0.23, 3.88) | 2.5  (0.32, 19.51) | 2.88  (0.37, 22.34) | 0.85  (0.11, 6.49) | Biologic-exposed patients |
| --- | --- | --- | --- | --- | --- | --- | --- | --- | --- | --- | --- | --- | --- |
| 1.24  (0.98, 1.57) | ADA  80/40 mg |  |  |  |  |  |  | 1.79  (0.24, 13.4) | 0.75  (0.19, 3.03) | 2  (0.26, 15.35) | 2.3  (0.3, 17.49) | 0.68  (0.09, 5.14) |  |
| 0.93  (0.76, 1.14) | 0.75  (0.55, 1) | ETR  105 mg |  |  |  |  |  |  | 0.42  (0.1, 1.76) | 1.12  (0.14, 8.78) | 1.29  (0.16, 10.05) | 0.38  (0.05, 2.97) |  |
| 0.9  (0.64, 1.27) | 0.73  (0.49, 1.07) | 0.97  (0.68, 1.4) | GOL  200/100 mg |  |  |  |  |  |  | 2.66  (0.61, 11.68) | 3.06  (0.71, 13.58) | 0.9  (0.21, 3.95) |  |
| 0.85  (0.6, 1.18) | 0.68  (0.47, 1) | 0.91  (0.64, 1.31) | 0.94  (0.72, 1.21) | GOL  400/200 mg |  |  |  |  |  |  | 1.15  (0.28, 4.71) | 0.34  (0.04, 2.73) |  |
| 0.7  (0.54, 0.91) | 0.57  (0.41, 0.78) | 0.76  (0.58, 0.99) | 0.78  (0.54, 1.13) | 0.83  (0.58, 1.2) | INF  10 mg/kg |  |  |  |  |  |  | 0.29  (0.04, 2.33) |  |
| 0.69  (0.44, 1.08) | 0.56  (0.34, 0.91) | 0.74  (0.47, 1.16) | 0.76  (0.45, 1.28) | 0.81  (0.48, 1.37) | 0.98  (0.63, 1.54) | INF  3.5 mg/kg |  |  |  |  |  |  |  |
| 0.69  (0.54, 0.86) | 0.55  (0.41, 0.75) | 0.74  (0.59, 0.92) | 0.76  (0.53, 1.09) | 0.81  (0.57, 1.15) | 0.98  (0.81, 1.17) | 1  (0.66, 1.5) | INF  5 mg/kg |  |  |  |  |  |  |
| 0.81  (0.57, 1.15) | 0.66  (0.44, 0.97) | 0.87  (0.6, 1.28) | 0.9  (0.58, 1.37) | 0.96  (0.62, 1.46) | 1.16  (0.78, 1.69) | 1.18  (0.7, 2.01) | 1.18  (0.82, 1.71) | MIR  300 mg |  |  |  |  |  |
| 1.34  (1.14, 1.58) | 1.08  (0.84, 1.39) | 1.44  (1.17, 1.78) | 1.48  (1.11, 2.01) | 1.58  (1.18, 2.12) | 1.9  (1.53, 2.38) | 1.95  (1.25, 3) | 1.95 (1.61, 2.37) | 1.64  (1.22, 2.26) | Placebo |  |  |  |  |
| 0.8  (0.51, 1.25) | 0.64  (0.4, 1.04) | 0.86  (0.53, 1.38) | 0.88  (0.53, 1.49) | 0.94  (0.56, 1.58) | 1.14  (0.7, 1.83) | 1.16  (0.63, 2.13) | 1.17  (0.73, 1.86) | 0.98  (0.58, 1.65) | 0.6  (0.39, 0.9) | UST  130 mg |  |  |  |
| 0.85  (0.53, 1.34) | 0.68  (0.41, 1.1) | 0.91  (0.56, 1.47) | 0.94  (0.55, 1.56) | 1  (0.6, 1.67) | 1.2  (0.73, 1.98) | 1.23  (0.67, 2.27) | 1.23  (0.77, 2.01) | 1.04 (0.61, 1.79) | 0.6  (0.41, 0.97) | 1.06  (0.74, 1.53) | UST  6 mg/kg |  |  |
| 0.87  (0.48, 1.45) | 0.7  (0.37, 1.21) | 0.93  (0.51, 1.59) | 0.96  (0.5, 1.7) | 1.03  (0.53, 1.81) | 1.24  (0.67, 2.13) | 1.26  (0.61, 2.42) | 1.26  (0.69, 2.16) | 1.07  (0.55, 1.91) | 0.65  (0.36, 1.05) | 1.08  (0.53, 2.06) | 1.02  (0.5, 1.96) | VED  300 mg |  |
| Biologic-naive patients | | | | | | | | | | | | |  |

Relative risk with 95% confidence intervals in parentheses. Comparisons between columns and rows should be read from left to right. The blue boxes represent statistically significant

comparisons and the white boxes represent non-statistically significant comparisons.

Note: ADA, adalimumab; ETR, etrolizumab; GOL, golimumab; IFX, infliximab; UST, ustekinumab; VED, vedolizumab; MIR, mirikizumab.

**Supplementary table 6**  League Table for Total Adverse Events and Serious Adverse Events in Induction Therapy.

| ADA  160/160mg | 1.3  (0.41, 4.13) | 1.02  (0.22, 4.54) | 2.3  (0.57, 10.95) | 0.78  (0.13, 5.37) | 0.96  (0.17, 6.48) | 0.96  (0.18, 6.32) | 1.78  (0.5, 7.7) | 0.94  (0.17, 6.44) | 0.87  (0.15, 5.91) | 2.38  (0.35, 20.88) | 1.91  (0.32, 14.06) | Serious adverse events |
| --- | --- | --- | --- | --- | --- | --- | --- | --- | --- | --- | --- | --- |
| .01  (0.82, 1.25) | ADA  160/80mg | 0.79  (0.29, 2.04) | 1.77  (0.75, 4.86) | 0.6  (0.15, 2.71) | 0.74  (0.19, 3.31) | 0.74 (0.21, 3.15) | 1.38  (0.71, 3.1) | 0.72  (0.2, 3.24) | 0.67  (0.18, 3) | 1.84  (0.38, 11.31) | 1.47  (0.37, 7.28) |  |
| 9  (0.67, 1.2) | 0.89  (0.73, 1.08) | ADA  80/40mg | 2.27  (0.73, 8.21) | 0.76  (0.17, 4.05) | 0.94  (0.22, 4.88) | 0.94  (0.23, 4.69) | 1.75  (0.73, 5.13) | 0.93  (0.22, 4.81) | 0.85  (0.2, 4.41) | 2.36  (0.43, 16.5) | 1.86  (0.42, 10.63) |  |
| .07  (0.81, 1.41) | 1.06  (0.88, 1.27) | 1.19  (0.93, 1.51) | ETR  105mg | 0.34  (0.08, 1.5) | 0.42  (0.09, 1.79) | 0.42  (0.1, 1.71) | 0.78  (0.33, 1.77) | 0.41  (0.1, 1.75) | 0.38  (0.09, 1.6) | 1.03  (0.19, 6.39) | 0.83  (0.18, 3.95) |  |
| .06  (0.74, 1.53) | 1.05  (0.78, 1.42) | 1.18  (0.86, 1.63) | 1  (0.73, 1.34) | GOL  200/100mg | 1.23(0.33, 4.54) | 1.24  (0.23, 6.68) | 2.3  (0.67, 8.06) | 1.21  (0.22, 6.86) | 1.12  (0.2, 6.35) | 3.1  (0.45, 23.01) | 2.45  (0.42, 15.13) |  |
| .02  (0.71, 1.48) | 1.02  (0.75, 1.36) | 1.14  (0.83, 1.56) | 0.96  (0.7, 1.29) | 0.97  (0.74, 1.25) | GOL  400/200mg | 1  (0.19, 5.35) | 1.84  (0.56, 6.36) | 0.98  (0.18, 5.49) | 0.9  (0.17, 5.04) | 2.5  (0.37, 18.53) | 1.98  (0.35, 12.24) |  |
| 1.08  (0.76, 1.52) | 1.07  (0.81, 1.39) | 1.2  (0.89, 1.61) | 1.01  (0.76, 1.32) | 1.01  (0.72, 1.42) | 1.05  (0.75, 1.47) | MIR  300mg | 1.86  (0.59, 5.88) | 0.98  (0.19, 5.14) | 0.9  (0.17, 4.79) | 2.49  (0.39, 17.83) | 1.98  (0.37, 11.47) |  |
| 1.04  (0.8, 1.36) | 1.03  (0.89, 1.2) | 1.16  (0.95, 1.41) | 0.97  (0.83, 1.15) | 0.98  (0.76, 1.27) | 1.01  (0.79, 1.32) | 0.97  (0.78, 1.21) | Placebo | 0.53  (0.16, 1.76) | 0.49  (0.14, 1.63) | 1.33  (0.31, 6.66) | 1.06  (0.3, 4.01) |  |
| 1.21  (0.84, 1.73) | 1.2  (0.9, 1.59) | 1.35  (0.98, 1.84) | 1.14  (0.84, 1.51) | 1.14  (0.8, 1.63) | 1.18  (0.83, 1.68) | 1.13  (0.81, 1.56) | 1.16  (0.91, 1.48) | UST  130mg | 0.92  (0.26, 3.24) | 2.54  (0.38, 18.13) | 2.01  (0.36, 12.17) |  |
| .99  (0.7, 1.4) | 0.98  (0.74, 1.29) | 1.1  (0.81, 1.49) | 0.93  (0.69, 1.22) | 0.93  (0.66, 1.32) | 0.97  (0.68, 1.37) | 0.92  (0.67, 1.27) | 0.95  (0.75, 1.2) | 0.82  (0.64, 1.04) | UST  6mg/kg | 2.77  (0.41, 19.86) | 2.19  (0.39, 13.04) |  |
| 1.08  (0.72, 1.61) | 1.07  (0.75, 1.51) | 1.2  (0.83, 1.73) | 1.01  (0.71, 1.42) | 1.02  (0.68, 1.52) | 1.05  (0.71, 1.58) | 1.01  (0.69, 1.47) | 1.04  (0.76, 1.41) | 0.89  (0.6, 1.32) | 1.09  (0.75, 1.6) | VED  300mg | 0.79  (0.11, 5.9) |  |
| 0.82  (0.57, 1.16) | 0.81  (0.6, 1.07) | 0.91  (0.66, 1.24) | 0.77  (0.56, 1.01) | 0.77  (0.53, 1.09) | 0.8  (0.55, 1.13) | 0.76  (0.54, 1.05) | **0.79**  **(0.6, 0.99)** | **0.67**  **(0.47, 0.95)** | 0.83  (0.58, 1.15) | 0.76  (0.51, 1.12) | Visi  5ug/kg |  |
| Total adverse events | | | | | | | | | | | |  |

Relative risk with 95% confidence intervals in parentheses. Comparisons between columns and rows should be read from left to right. The blue boxes represent statistically significant

comparisons and the white boxes represent non-statistically significant comparisons.

Note: ADA, adalimumab; ETR, etrolizumab; GOL, golimumab; IFX, infliximab; UST, ustekinumab; VED, vedolizumab; MIR, mirikizumab; Visi, visilizumab.

**Supplementary table 7**  League table for infections and adverse events leading to withdrawal in induction therapy.

| ADA  160/160mg | 1.07  (0.1, 12.07) | 0.48  (0.01, 5.59) | 2.08  (0.11, 41.83) | 0.18  (0, 10.03) | 0.18  (0, 9.78) | 0.16  (0, 5.23) | 0.75  (0.04, 9.66) |  |  |  | 1.71  (0.03, 81.98) | Adverse events leading to withdrawal |
| --- | --- | --- | --- | --- | --- | --- | --- | --- | --- | --- | --- | --- |
| 0.89  (0.55, 1.44) | ADA  160/80mg | 0.44  (0.03, 1.79) | 1.92  (0.34, 11.76) | 0.17  (0, 4.7) | 0.17  (0, 4.64) | 0.15  (0.01, 2.02) | 0.69  (0.13, 2.35) |  |  |  | 1.58  (0.06, 36.97) |  |
| 0.81  (0.43, 1.57) | 0.91  (0.59, 1.45) | ADA  80/40mg | 4.6  (0.65, 97.01) | 0.41  (0.01, 22.68) | 0.42  (0.01, 22.81) | 0.34  (0.02, 11.91) | 1.57  (0.33, 16.8) |  |  |  | 3.74  (0.19, 188.56) |  |
| 0.91  (0.48, 1.67) | 1.02  (0.68, 1.51) | 1.13  (0.65, 1.88) | ETR  105mg | 0.09  (0, 2.87) | 0.09  (0, 2.82) | 0.08  (0, 1.2) | 0.35  (0.05, 1.67) |  |  |  | 0.82  (0.03, 22.18) |  |
| 0.87  (0.39, 1.98) | 0.98  (0.51, 1.88) | 1.08  (0.51, 2.2) | 0.96  (0.49, 1.87) | GOL  200/100mg | 1.01  (0.02, 62.98) | 0.86  (0.02, 83.72) | 3.91  (0.17, 197.79) |  |  |  | 9.62  (0.15, 1219.97) |  |
| 0.83  (0.37, 1.84) | 0.93  (0.49, 1.77) | 1.03  (0.49, 2.08) | 0.91  (0.48, 1.77) | 0.95  (0.55, 1.66) | GOL  400/200mg | 0.85  (0.02, 87.08) | 3.86  (0.17, 209.26) |  |  |  | 9.67  (0.14, 1277.98) |  |
| 0.77  (0.36, 1.65) | 0.87  (0.48, 1.57) | 0.96  (0.49, 1.82) | 0.85  (0.47, 1.56) | 0.89  (0.43, 1.85) | 0.93  (0.45, 1.94) | MIR  300mg | 4.61  (0.4, 52.79) |  |  |  | 10.74  (0.28, 487.87) |  |
| 0.84  (0.47, 1.52) | 0.95  (0.68, 1.33) | 1.04  (0.66, 1.62) | 0.93  (0.66, 1.33) | 0.97  (0.55, 1.7) | 1.02  (0.59, 1.77) | 1.09  (0.67, 1.78) | Placebo |  |  |  | 2.31  (0.16, 46.35) |  |
| 0.82  (0.38, 1.79) | 0.92  (0.5, 1.7) | 1.02  (0.51, 1.98) | 0.9  (0.49, 1.7) | 0.94  (0.44, 2.02) | 0.99  (0.47, 2.11) | 1.06  (0.52, 2.14) | 0.97  (0.58, 1.62) | UST  130mg |  |  |  |  |
| 0.82  (0.38, 1.78) | 0.92  (0.5, 1.7) | 1.02  (0.51, 1.98) | 0.9  (0.49, 1.69) | 0.95  (0.44, 2.02) | 0.98  (0.47, 2.09) | 1.06  (0.52, 2.13) | 0.97  (0.58, 1.62) | 1  (0.6, 1.67) | UST  6mg/kg |  |  |  |
| 0.72  (0.29, 1.73) | 0.81  (0.38, 1.67) | 0.89  (0.39, 1.93) | 0.79  (0.37, 1.67) | 0.83  (0.35, 1.94) | 0.87  (0.36, 2.01) | 0.93  (0.4, 2.06) | 0.86  (0.43, 1.62) | 0.88  (0.37, 2.01) | 0.88  (0.37, 2) | Visi  5ug/kg |  |  |
|  |  |  |  |  |  |  |  |  |  |  | VED  300mg |  |
| Infections | | | | | | | | | | | |  |

Relative risk with 95% confidence intervals in parentheses. Comparisons between columns and rows should be read from left to right. The blue boxes represent statistically significant

comparisons and the white boxes represent non-statistically significant comparisons.

Note: ADA, adalimumab; ETR, etrolizumab; GOL, golimumab; IFX, infliximab; UST, ustekinumab; VED, vedolizumab; MIR, mirikizumab.

**Supplementary table 8**  League table for total adverse events and serious adverse events in maintenance therapy.

| ADA  40mgEOW | 0.99  (0.51, 1.91) | 1.17  (0.53, 2.52) | 1.26  (0.45, 3.02) | 0.83  (0.29, 2.26) | 0.67  (0.3, 1.35) | 0.41  (0.05, 1.94) | 0.68  (0.33, 1.32) | 0.39  (0.13, 1.1) | 0.92  (0.53, 1.54) | 0.71  (0.25, 1.93) | 0.8  (0.29, 2.17) | 0.72  (0.26, 2.03) | 0.86  (0.52, 1.49) | Serious adverse events |
| --- | --- | --- | --- | --- | --- | --- | --- | --- | --- | --- | --- | --- | --- | --- |
| 0.99  (0.83, 1.19) | ADA  40mgQW | 1.18  (0.42, 3.24) | 1.28  (0.37, 3.72) | 0.84  (0.24, 2.76) | 0.68  (0.24, 1.76) | 0.41  (0.05, 2.24) | 0.68  (0.26, 1.74) | 0.4  (0.11, 1.34) | 0.92  (0.39, 2.14) | 0.72  (0.21, 2.37 | 0.81  (0.24, 2.65) | 0.73  (0.21, 2.49 | 0.87  (0.39, 2.08) |  |
| 1.08  (0.89, 1.33) | 1.09  (0.84, 1.43) | ETR  105mg | 1.08  (0.39, 2.73) | 0.71  (0.24, 2) | 0.57  (0.28, 1.14) | 0.35  (0.04, 1.65) | 0.58  (0.32, 1.02) | 0.34  (0.11, 0.95) | 0.79  (0.45, 1.38) | 0.61  (0.21, 1.7) | 0.68  (0.24, 1.9) | 0.62  (0.21, 1.87) | 0.74  (0.37, 1.57) |  |
| 0.89  (0.7, 1.1) | 0.9  (0.66, 1.18) | 0.82  (0.66, 0.99) | GOL  100mg | 0.66  (0.29, 1.59) | 0.53  (0.22, 1.4) | 0.32  (0.04, 1.8) | 0.54  (0.23, 1.38) | 0.31  (0.1, 1.08 | 0.73  (0.35, 1.67) | 0.56  (0.18, 1.92) | 0.63  (0.21, 2.12) | 0.57  (0.17, 2.06) | 0.69  (0.3, 1.87) |  |
| 0.93  (0.71, 1.19) | 0.94  (0.68, 1.27) | 0.86  (0.67, 1.07) | 1.04  (0.86, 1.27) | GOL  50mg | 0.81  (0.29, 2.25) | 0.48  (0.06, 2.81) | 0.81  (0.31, 2.21 | 0.47  (0.14, 1.68) | 1.1  (0.47, 2.72) | 0.85  (0.25, 2.97) | 0.96  (0.29, 3.32 | 0.87  (0.24, 3.21) | 1.05  (0.4, 2.97) |  |
| 1  (0.81, 1.22) | 1.01  (0.77, 1.32) | 0.93  (0.78, 1.08) | 1.13  (0.92, 1.41) | 1.08  (0.85, 1.38) | INF  10mg/kg | 0.61  (0.08, 2.87) | 1.01  (0.6, 1.74) | 0.59  (0.2, 1.66) | 1.37  (0.84, 2.36) | 1.06  (0.39, 2.93) | 1.19  (0.44, 3.28) | 1.08  (0.38, 3.21) | 1.29  (0.69, 2.74) |  |
| 1.09  (0.67, 1.84) | 1.1  (0.66, 1.91) | 1  (0.63, 1.68) | 1.23  (0.76, 2.1) | 1.18  (0.72, 2.03) | 1.09  (0.68, 1.82) | INF  3.5mg/kg | 1.65  (0.37, 12.05) | 0.96  (0.17, 8.54) | 2.24  (0.51, 16.16) | 1.75  (0.31, 14.9) | 1.98  (0.35, 16.96) | 1.82  (0.3, 16.2) | 2.13  (0.45, 16.37) |  |
| 1.03  (0.84, 1.24) | 1.04  (0.79, 1.34) | 0.95  (0.82, 1.08) | 1.16  (0.95, 1.43) | 1.11  (0.88, 1.41) | 1.03  (0.9, 1.17) | 0.95  (0.57, 1.49) | INF  5mg/kg | 0.58  (0.21, 1.56) | 1.35  (0.88, 2.15) | 1.05  (0.39, 2.78) | 1.19  (0.45, 3.09) | 1.07  (0.38, 3.07) | 1.28  (0.71, 2.54) |  |
| 1.13  (0.87, 1.44) | 1.14  (0.83, 1.54) | 1.04  (0.82, 1.3) | 1.27  (0.99, 1.65) | 1.22  (0.92, 1.62) | 1.13  (0.89, 1.42) | 1.04  (0.6, 1.69) | 1.09  (0.87, 1.38) | MIR  200mg | 2.35  (0.96, 5.93) | 1.81  (0.51, 6.53) | 2.04  (0.59, 7.4) | 1.84  (0.51, 7) | 2.22  (0.84, 6.27) |  |
| 1.06  (0.9, 1.24) | 1.07  (0.84, 1.35) | 0.98  (0.86, 1.1) | 1.19  (1.02, 1.42) | 1.15  (0.94, 1.41) | 1.06  (0.93, 1.21) | 0.98  (0.59, 1.53) | 1.03  (0.92, 1.16) | 0.94  (0.77, 1.15) | Placebo | 0.77  (0.32, 1.84) | 0.87  (0.37, 2.03) | 0.79  (0.31, 2.02) | 0.95  (0.62, 1.54) |  |
| 1.21  (0.94, 1.56) | 1.22  (0.89, 1.66) | 1.12  (0.88, 1.4) | 1.36  (1.06, 1.78) | 1.31  (0.99, 1.74) | 1.21  (0.95, 1.54) | 1.11  (0.65, 1.82) | 1.17  (0.94, 1.48) | 1.07  (0.81, 1.42) | 1.14  (0.93, 1.39) | UST  90mg Q12W | 1.13  (0.47, 2.8) | 1.02  (0.28, 3.72) | 1.23  (0.47, 3.39) |  |
| 1.09  (0.84, 1.38) | 1.09  (0.8, 1.47) | 1  (0.79, 1.25) | 1.22  (0.95, 1.58) | 1.17  (0.89, 1.55) | 1.08  (0.86, 1.36) | 1  (0.58, 1.62) | 1.05  (0.84, 1.32) | 0.96  (0.73, 1.26) | 1.02  (0.84, 1.24) | 0.89  (0.73, 1.09) | UST  90mg Q8W | 0.89  (0.25, 3.34) | 1.08  (0.43, 2.94) |  |
| 1.24  (0.95, 1.62) | 1.26  (0.91, 1.71) | 1.15  (0.88, 1.49) | 1.4  (1.06, 1.86) | 1.34  (0.99, 1.82) | 1.24  (0.95, 1.62) | 1.14  (0.66, 1.91) | 1.21  (0.94, 1.56) | 1.1  (0.82, 1.49) | 1.17  (0.93, 1.48) | 1.03  (0.76, 1.39) | 1.15  (0.85, 1.55) | VED  108mg | 1.21  (0.48, 3.17) |  |
| 1.06  (0.9, 1.22) | 1.07  (0.84, 1.34) | 0.98  (0.82, 1.14) | 1.19  (0.98, 1.46) | 1.14  (0.91, 1.44) | 1.06  (0.89, 1.25) | 0.97  (0.58, 1.55) | 1.03  (0.87, 1.2) | 0.94  (0.75, 1.18) | 1  (0.89, 1.11) | 0.87  (0.69, 1.1) | 0.98  (0.78, 1.22) | 0.85  (0.67, 1.07) | VED  300mg |  |
| Total adverse events | | | | | | | | | | | | | |  |

Relative risk with 95% confidence intervals in parentheses. Comparisons between columns and rows should be read from left to right. The blue boxes represent statistically significant

comparisons and the white boxes represent non-statistically significant comparisons.

Note: ADA, adalimumab; ETR, etrolizumab; GOL, golimumab; IFX, infliximab; UST, ustekinumab; VED, vedolizumab; MIR, mirikizumab; EOW, every other week; QW, every week; Q12W, every 12weeks; Q8W, every 8 weeks.

**Supplementary table 9**  League table for infections and adverse events leading to withdrawal in maintenance therapy.

| ADA  40 mg EOW | 0.86  (0.23, 3.17) | 1  (0.22, 3.87) | 2.01  (0.31, 11.82) | 1.12  (0.16, 6.9) | 0.98  (0.2, 4.13) | 0.77  (0.15, 2.8) | 0.25  (0.04, 1.54) | 1.4  (0.44, 4.02) | 0.62  (0.1, 3.56) | 0.32  (0.05, 1.99) | 0.28  (0.01, 3.85) | 0.72  (0.24, 2.33) | Adverse Events Leading to Withdrawal |
| --- | --- | --- | --- | --- | --- | --- | --- | --- | --- | --- | --- | --- | --- |
| 1.03  (0.76, 1.4) | ADA  40 mg QW | 1.17  (0.15, 7.47) | 2.35  (0.24, 20.54) | 1.31  (0.12, 11.96) | 1.15  (0.14, 7.74) | 0.9  (0.11, 5.28) | 0.29  (0.03, 2.71) | 1.63  (0.28, 8.5) | 0.72  (0.07, 6.36) | 0.38  (0.04, 3.45) | 0.32  (0.01, 5.99) | 0.84  (0.16, 4.93) |  |
| 0.97  (0.66, 1.44) | 0.94  (0.58, 1.54) | ETR  105 mg | 2  (0.37, 11.55) | 1.12  (0.2, 6.77) | 0.98  (0.28, 3.47) | 0.76  (0.26, 1.96) | 0.25  (0.04, 1.49) | 1.4  (0.58, 3.65) | 0.62  (0.12, 3.51) | 0.32  (0.05, 1.98) | 0.28  (0.01, 4.27) | 0.72  (0.18, 3.55) |  |
| 0.76  (0.48, 1.16) | 0.73  (0.42, 1.23) | 0.78  (0.51, 1.14) | GOL  100 mg | 0.56  (0.13, 2.42) | 0.49  (0.08, 2.79) | 0.38  (0.06, 1.92) | 0.12  (0.02, 0.98) | 0.7  (0.16, 2.94) | 0.31  (0.04, 2.34) | 0.16  (0.02, 1.33) | 0.14  (0, 2.58) | 0.36  (0.06, 2.47) |  |
| 0.78  (0.48, 1.27) | 0.76  (0.43, 1.34) | 0.81  (0.52, 1.26) | 1.04  (0.74, 1.49) | GOL  50 mg | 0.87  (0.14, 5.44) | 0.68  (0.11, 3.74) | 0.22  (0.03, 1.89) | 1.24  (0.28, 5.8) | 0.55  (0.07, 4.49) | 0.29  (0.03, 2.53) | 0.25  (0.01, 4.87) | 0.65  (0.1, 4.8) |  |
| 0.94  (0.62, 1.41) | 0.91  (0.55, 1.51) | 0.96  (0.69, 1.34) | 1.24  (0.82, 1.91) | 1.19  (0.75, 1.91) | INF  10 mg/kg | 0.78  (0.25, 2.16) | 0.25  (0.04, 1.59) | 1.42  (0.53, 4.1) | 0.62  (0.11, 3.8) | 0.33  (0.05, 2.12) | 0.28  (0.01, 4.51) | 0.74  (0.17, 3.86) |  |
| 1.04  (0.7, 1.54) | 1.01  (0.61, 1.64) | 1.07  (0.81, 1.4) | 1.38  (0.93, 2.09) | 1.33  (0.85, 2.09) | 1.11  (0.85, 1.46) | INF  5 mg/kg | 0.33  (0.06, 2.1) | 1.82  (0.79, 5.14) | 0.8  (0.16, 4.93) | 0.43  (0.07, 2.78) | 0.37  (0.01, 5.83) | 0.95  (0.25, 5.01) |  |
| 1.08  (0.64, 1.77) | 1.05  (0.59, 1.85) | 1.11  (0.69, 1.75) | 1.43  (0.86, 2.4) | 1.38  (0.78, 2.37) | 1.15  (0.7, 1.87) | 1.03  (0.64, 1.66) | MIR  200 mg | 5.62  (1.26, 26.29) | 2.48  (0.32, 19.91) | 1.29  (0.15, 11.12) | 1.12  (0.03, 21.54) | 2.92  (0.47, 21.64) |  |
| 1.14  (0.84, 1.55) | 1.11  (0.72, 1.69) | 1.17  (0.92, 1.49) | 1.51  (1.11, 2.11) | 1.46  (1, 2.12) | 1.22  (0.93, 1.61) | 1.1  (0.86, 1.41) | 1.06  (0.72, 1.6) | Placebo | 0.44  (0.11, 1.83) | 0.23  (0.05, 1.05) | 0.2  (0.01, 2.54) | 0.52  (0.17, 1.77) |  |
| 1.57  (0.97, 2.52) | 1.52  (0.86, 2.64) | 1.62  (1.05, 2.47) | 2.07  (1.31, 3.38) | 2  (1.2, 3.37) | 1.68  (1.07, 2.62) | 1.51  (0.98, 2.32) | 1.46  (0.86, 2.5) | 1.38  (0.96, 1.96) | UST 90 mg Q12W | 0.53  (0.1, 2.55) | 0.45  (0.01, 8.24) | 1.17  (0.2, 7.92) |  |
| 1.08  (0.69, 1.68) | 1.05  (0.61, 1.79) | 1.11  (0.74, 1.65) | 1.43  (0.92, 2.27) | 1.38  (0.85, 2.27) | 1.15  (0.76, 1.76) | 1.04  (0.69, 1.56) | 1  (0.61, 1.69) | 0.95  (0.68, 1.3) | 0.69  (0.48, 0.97) | UST 90 mg Q8W | 0.86  (0.02, 16.97) | 2.24  (0.36, 17.05) |  |
| 1.47  (0.79, 2.83) | 1.43  (0.72, 2.91) | 1.52  (0.84, 2.8) | 1.95  (1.05, 3.81) | 1.88  (0.98, 3.73) | 1.58  (0.86, 2.98) | 1.42  (0.78, 2.62) | 1.37  (0.71, 2.72) | 1.29  (0.75, 2.29) | 0.94  (0.49, 1.83) | 1.36  (0.73, 2.63) | VED  108 mg | 2.61  (0.21, 89.54) |  |
| 1.07  (0.72, 1.58) | 1.03  (0.63, 1.68) | 1.1  (0.78, 1.54) | 1.41  (0.96, 2.13) | 1.36  (0.87, 2.12) | 1.14  (0.79, 1.64) | 1.03  (0.72, 1.45) | 0.99  (0.63, 1.6) | 0.94  (0.73, 1.19) | 0.68  (0.44, 1.04) | 0.99  (0.66, 1.47) | 0.72  (0.41, 1.24) | VED  300 mg |  |
| Infections | | | | | | | | | | | | |  |

Relative risk with 95% confidence intervals in parentheses. Comparisons between columns and rows should be read from left to right. The blue boxes represent statistically significant

comparisons and the white boxes represent non-statistically significant comparisons.

Note: ADA, adalimumab; ETR, etrolizumab; GOL, golimumab; IFX, infliximab; UST, ustekinumab; VED, vedolizumab; MIR, mirikizumab; EOW, every other week; QW, every week; Q12W, every 12weeks; Q8W, every 8 weeks; Q4W, every 4 weeks.

**Supplementary table 10**  PRISMA checklist

| **Section and Topic** | **Item #** | **Checklist item** | **Location where item is reported** |
| --- | --- | --- | --- |
| **TITLE** | | |  |
| Title | 1 | Identify the report as a systematic review. | Title |
| **ABSTRACT** | | |  |
| Abstract | 2 | See the PRISMA 2020 for Abstracts checklist. | Abstract |
| **INTRODUCTION** | | |  |
| Rationale | 3 | Describe the rationale for the review in the context of existing knowledge. | Introduction |
| Objectives | 4 | Provide an explicit statement of the objective(s) or question(s) the review addresses. | Introduction |
| **METHODS** | | |  |
| Eligibility criteria | 5 | Specify the inclusion and exclusion criteria for the review and how studies were grouped for the syntheses. | Methods |
| Information sources | 6 | Specify all databases, registers, websites, organisations, reference lists and other sources searched or consulted to identify studies. Specify the date when each source was last searched or consulted. | Methods |
| Search strategy | 7 | Present the full search strategies for all databases, registers and websites, including any filters and limits used. | Supplementary 1 |
| Selection process | 8 | Specify the methods used to decide whether a study met the inclusion criteria of the review, including how many reviewers screened each record and each report retrieved, whether they worked independently, and if applicable, details of automation tools used in the process. | Methods |
| Data collection process | 9 | Specify the methods used to collect data from reports, including how many reviewers collected data from each report, whether they worked independently, any processes for obtaining or confirming data from study investigators, and if applicable, details of automation tools used in the process. | Methods |
| Data items | 10a | List and define all outcomes for which data were sought. Specify whether all results that were compatible with each outcome domain in each study were sought (e.g. for all measures, time points, analyses), and if not, the methods used to decide which results to collect. | Methods |
|  | 10b | List and define all other variables for which data were sought (e.g. participant and intervention characteristics, funding sources). Describe any assumptions made about any missing or unclear information. | Methods |
| Study risk of bias assessment | 11 | Specify the methods used to assess risk of bias in the included studies, including details of the tool(s) used, how many reviewers assessed each study and whether they worked independently, and if applicable, details of automation tools used in the process. | Methods |
| Effect measures | 12 | Specify for each outcome the effect measure(s) (e.g. risk ratio, mean difference) used in the synthesis or presentation of results. | Methods |
| Synthesis methods | 13a | Describe the processes used to decide which studies were eligible for each synthesis (e.g. tabulating the study intervention characteristics and comparing against the planned groups for each synthesis (item #5)). | Methods |
|  | 13b | Describe any methods required to prepare the data for presentation or synthesis, such as handling of missing summary statistics, or data conversions. | NA |
|  | 13c | Describe any methods used to tabulate or visually display results of individual studies and syntheses. | NA |
|  | 13d | Describe any methods used to synthesize results and provide a rationale for the choice(s). If meta-analysis was performed, describe the model(s), method(s) to identify the presence and extent of statistical heterogeneity, and software package(s) used. | Methods |
|  | 13e | Describe any methods used to explore possible causes of heterogeneity among study results (e.g. subgroup analysis, meta-regression). | Methods |
|  | 13f | Describe any sensitivity analyses conducted to assess robustness of the synthesized results. | NA |
| Reporting bias assessment | 14 | Describe any methods used to assess risk of bias due to missing results in a synthesis (arising from reporting biases). | Methods |
| Certainty assessment | 15 | Describe any methods used to assess certainty (or confidence) in the body of evidence for an outcome. | NA |
| **RESULTS** | | |  |
| Study selection | 16a | Describe the results of the search and selection process, from the number of records identified in the search to the number of studies included in the review, ideally using a flow diagram. | Results;  Figure 1 |
|  | 16b | Cite studies that might appear to meet the inclusion criteria, but which were excluded, and explain why they were excluded. | NA |
| Study characteristics | 17 | Cite each included study and present its characteristics. | Supplementary table 2 |
| Risk of bias in studies | 18 | Present assessments of risk of bias for each included study. | Results;  Supplementary figure 1 |
| Results of individual studies | 19 | For all outcomes, present, for each study: (a) summary statistics for each group (where appropriate) and (b) an effect estimate and its precision (e.g. confidence/credible interval), ideally using structured tables or plots. | Supplementary table 1 |
| Results of syntheses | 20a | For each synthesis, briefly summarise the characteristics and risk of bias among contributing studies. | Results |
|  | 20b | Present results of all statistical syntheses conducted. If meta-analysis was done, present for each the summary estimate and its precision (e.g. confidence/credible interval) and measures of statistical heterogeneity. If comparing groups, describe the direction of the effect. | Results |
|  | 20c | Present results of all investigations of possible causes of heterogeneity among study results. | NA |
|  | 20d | Present results of all sensitivity analyses conducted to assess the robustness of the synthesized results. | NA |
| Reporting biases | 21 | Present assessments of risk of bias due to missing results (arising from reporting biases) for each synthesis assessed. | NA |
| Certainty of evidence | 22 | Present assessments of certainty (or confidence) in the body of evidence for each outcome assessed. | NA |
| **DISCUSSION** | | |  |
| Discussion | 23a | Provide a general interpretation of the results in the context of other evidence. | Discussion |
|  | 23b | Discuss any limitations of the evidence included in the review. | Discussion |
|  | 23c | Discuss any limitations of the review processes used. | Discussion |
|  | 23d | Discuss implications of the results for practice, policy, and future research. | Discussion |
| **OTHER INFORMATION** | | |  |
| Registration and protocol | 24a | Provide registration information for the review, including register name and registration number, or state that the review was not registered. | Methods |
|  | 24b | Indicate where the review protocol can be accessed, or state that a protocol was not prepared. | Methods |
|  | 24c | Describe and explain any amendments to information provided at registration or in the protocol. | NA |
| Support | 25 | Describe sources of financial or non-financial support for the review, and the role of the funders or sponsors in the review. | Acknowledgments |
| Competing interests | 26 | Declare any competing interests of review authors. | Authors’ contributions |
| Availability of data, code and other materials | 27 | Report which of the following are publicly available and where they can be found: template data collection forms; data extracted from included studies; data used for all analyses; analytic code; any other materials used in the review. | Data availability |
